# *Dazl* is a target RNA suppressed by mammalian NANOS2 in sexually differentiating male germ cells

**DOI:** 10.1038/ncomms11272

**Published:** 2016-04-13

**Authors:** Yuzuru Kato, Takeo Katsuki, Hiroki Kokubo, Aki Masuda, Yumiko Saga

**Affiliations:** 1 Division of Mammalian Development, Genetic Strains Research Center, National Institute of Genetics, Yata 1111, Mishima, Shizuoka 411-8540, Japan; 2 Department of Genetics, SOKENDAI, Yata 1111, Mishima, Shizuoka 411-8540, Japan; 3 Kavli Institute for Brain and Mind, University of California, San Diego, 9500 Gilman Drive, La Jolla, California 92093, USA; 4 Department of Biological Sciences, Graduate School of Science, The University of Tokyo, Hongo 7-3-1, Bunkyo-ku, Tokyo 113-0033, Japan; 5 Present address: Department of Cardiovascular Physiology and Medicine, Graduate School of Biomedical and Health Sciences, Hiroshima University, Kasumi 1-2-3, Minami-ku, Hiroshima 734-8551, Japan

## Abstract

Evolutionally conserved Nanos RNA-binding proteins play crucial roles in germ cell development. While a mammalian Nanos family protein, NANOS2, is required for sexual differentiation of male (XY) germ cells in mice, the underlying mechanisms and the identities of its target RNAs *in vivo* remain elusive. Using comprehensive microarray analysis and a bacterial artificial chromosome transgenic system, here we identify *Dazl*, a germ cell-specific gene encoding an RNA-binding protein implicated in translation, as a crucial target of NANOS2. Importantly, removal of the *Dazl* 3′-untranslated region in XY germ cells stabilizes the *Dazl* mRNA, resulting in elevated meiotic gene expression, abnormal resumption of the cell cycle and impaired processing-body formation, reminiscent of *Nanos2*-knockout phenotypes. Furthermore, our data suggest that NANOS2 acts as an antagonist of the DAZL protein. We propose a dual system of NANOS2-mediated suppression of *Dazl* expression as a pivotal molecular mechanism promoting sexual differentiation of XY germ cells.

## Abstract (web summary)

The Nanos family protein, NANOS2, is required for sexual differentiation of male germ cells in mice, however, the molecular RNA targets are unknown. Here, Kato *et al*. identify *Dazl*, a germ cell-specific gene, as being a target of NANOS, with stabilized *Dazl* mRNA causing abnormal resumption of the cell cycle.

## Graphical abstract

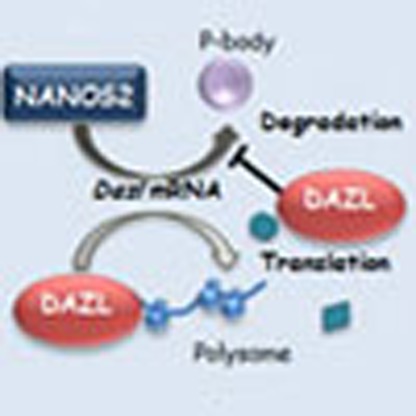

Germ cells are specialized to transmit genetic information to the next generation by undergoing a discrete developmental process from the somatic lineage. While the strategy varies among model organisms, for instance, the germ cell lineage is determined by maternally inherited cytoplasmic granules in *Caenorhabditis elegans* and *Drosophila melanogaster* but by an inductive signal in the mouse^1^,^2^,^3^,^4^, a common feature of germ cell development is the participation of evolutionally conserved RNA-binding proteins^5^. *Nanos* is one of the genes essential for germ cell development across species; it is shown that Nanos proteins are required for meiosis initiation and the sperm–oocyte switch in *C. elegans*^6^,^7^, and for germ cell formation and the maintenance of ovarian germline stem cells in *Drosophila*^8^,^9^. The mammalian genome contains three *Nanos* genes (*Nanos1, 2* and *3*), among which *Nanos2* and *Nanos3* are indispensable for germ cell development in mice^10^,^11^. Although both NANOS2 and NANOS3 are implicated in RNA degradation^12^,^13^,^14^, their target RNAs remain elusive.

*Nanos2* is a male-specific gene, expression of which begins once primordial germ cells (PGCs) enter the sex-specific process of sexual differentiation^10^. In mice, sexual differentiation of PGCs takes place in developing gonads after embryonic day (E) 11.5, at which point sexually dimorphic PGCs enter meiosis and mitotic quiescence, in XX and XY gonads, respectively^15^. While initiation of meiosis in XX gonads is triggered by retinoic acid (RA) signalling, which stimulates the *Stra8* gene expression required for premeiotic DNA replication in XX germ cells^16^,^17^,^18^, RA signalling is suppressed by RA-metabolizing enzyme, CYP26b1, in sertoli cells and XY germ cells cease proliferation at the G0/G1 phase in XY gonads^16^,^17^,^19^.

We previously showed that NANOS2 plays an indispensable role in achieving mitotic quiescence in XY germ cells; in *Nanos2*-deficient mice, XY germ cells abnormally enter meiosis in embryonic gonads^20^. Because NANOS2 negatively regulates its target via RNA degradation through forming a protein complex with CCR4-NOT deadenylase^10^,^12^,^13^, it is supposed that NANOS2 suppresses the level of certain target RNAs involved in meiosis. A putative target is *Stra8*, as it is required for the initiation of meiosis and its strong upregulation in *Nanos2*-deficient XY germ cells^18^,^20^. However, we recently showed that additional deletion of *Stra8* in a *Nanos2*-deficient background could not rescue the defective male-specific gene expression and resumption of mitotic cell cycles, despite successful suppression of meiosis in *Nanos2* and *Stra8* double-deficient XY germ cells^21^. Therefore, how NANOS2 contributes to sexual differentiation of XY germ cells, particularly, which target NANOS2 regulates *in vivo*, remains unknown.

In this study, we aimed to determine NANOS2 target RNAs involved in sexual differentiation of XY germ cells. By conducting comprehensive microarray analysis, we identify the *Dazl* mRNA as a strong candidate for a NANOS2 target. Using a bacterial artificial chromosome (BAC) transgenic mouse system, we demonstrate that NANOS2 represses *Dazl* expression in sexually differentiating XY germ cells. Furthermore, our data suggest that NANOS2 acts as an antagonist of DAZL for common target RNAs. We propose that NANOS2 uses dual mechanisms for suppressing *Dazl* expression to promote sexual differentiation of XY germ cells.

## Results

### NANOS2 post-transcriptionally represses *Dazl* expression

**Figure 1:**
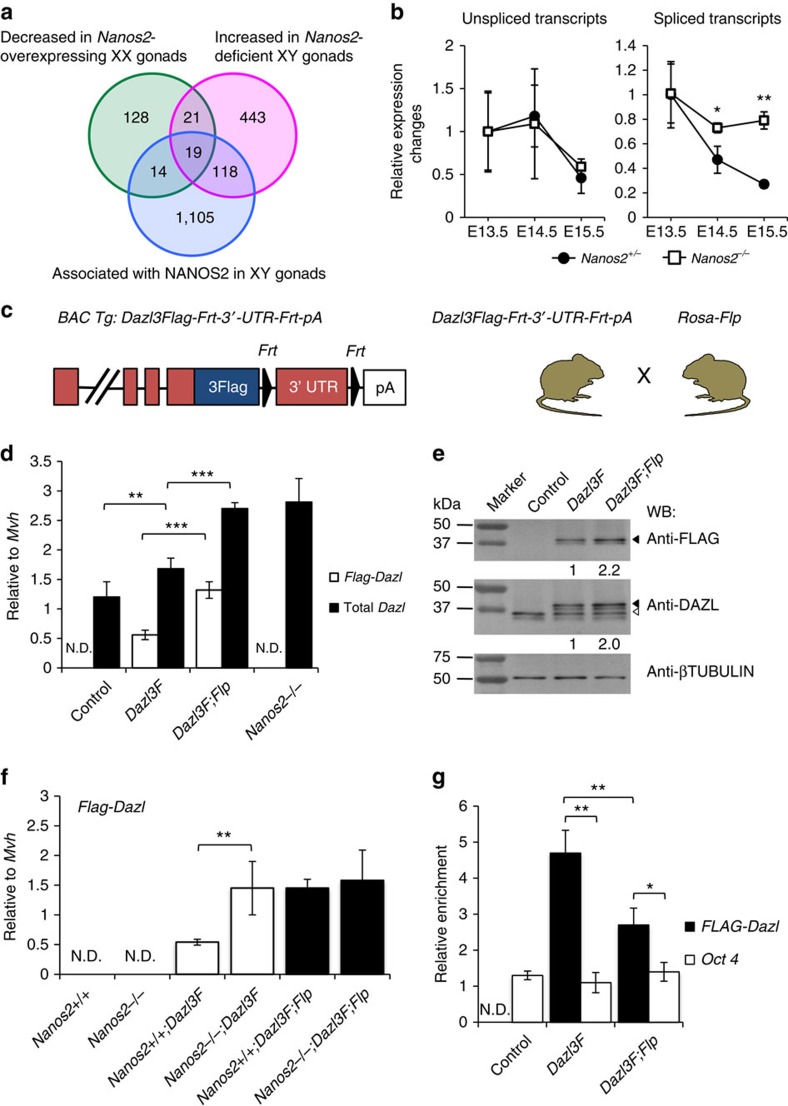
NANOS2 post-transcriptionally suppresses *Dazl* via association with its 3′-UTR. (**a**) Venn diagrams show the overlap among gene probes for mRNAs that were decreased more than 1.5-fold in *Nanos2*-expressing XX gonads compared with control XX gonads at E11.5 (green), that were increased more than twofold in *Nanos2*^−/−^ XY gonads compared with *Nanos2*^+/−^ XY gonads at E14.5 or E15.5 (pink), and that were associated with NANOS2 protein at a fourfold higher level than the input at E14.5 (blue). (**b**) RT-qPCR analysis of unspliced and spliced transcripts of *Dazl* in XY gonads. The expression level was normalized against the murine *vasa* homologue. The value for *Nanos2*^+/−^ at E13.5 was set as the standard (*n*=3–4). (**c**) The transgenic (Tg) BAC construct. (**d**) RT-qPCR analysis of *Flag-Dazl* (white bars) and total *Dazl* (black bars) expression levels in XY gonads at E15.5 (*n*=4–7). The *y* axis shows the expression level relative to *Mvh*. (**e**) WB analysis of FLAG-DAZL and endogenous DAZL proteins in XY gonads at E15.5; β-TUBULIN was used as a control. Filled and open arrowheads indicate FLAG-DAZL and endogenous DAZL protein, respectively. Relative signal intensity of FLAG-DAZL protein was indicated at the bottom of each panel. (**f**) *Flag-Dazl* expression in *Nanos2*^*+/+*^ and *Nanos2*^−/−^ gonads at E15.5 (*n*=3–5). The *y* axis shows the expression level relative to *Mvh*. (**g**) RNA-immunoprecipitation and RT-qPCR analysis, using anti-NANOS2 antibody, of XY gonads at E15.5. The *y* axis represents the immunoprecipitate/input ratio normalized against that of *Gapdh* (*n*=4). *Oct4* was used as a negative control. (**b**,**d**,**f**,**g**) Error bars, ±s.d. Significance levels of changes are indicated (two-tailed Student's *t*-test; ***P<0.0005, **P<0.005, *P<0.05). N.D., not detected.

We previously reported putative NANOS2 targets identified by overlapping the microarray data of genes whose mRNA levels were increased in *Nanos2*^−/−^ XY gonads and of NANOS2-associated mRNAs^21^. To further narrow down the candidates, we conducted an additional microarray analysis of RNAs from *Nanos2*-expressing XX gonads (Supplementary Fig. 1a). FLAG-tagged NANOS2 was induced in more than 70% of XX germ cells by injecting tamoxifen at E10.5 into a *CAG-CAT*^*flox*^*-3FlagNanos2-pA* mouse^20^ crossed with *Oct4-CreER*^*T2*^*-pA* (ref. 22). We confirmed that meiosis was successfully prevented in the NANOS2-expressing XX germ cells (Supplementary Fig. 1b and c) as previously reported^20^,^22^. We selected 182 probes that were downregulated upon the induction of *Nanos2* at E11.5, and looked for overlaps with our previous microarray data^21^ (Fig. 1a). We identified 19 probes (16 genes) that fulfilled all three of the following criteria: they were upregulated in *Nanos2*-deficient XY gonads; downregulated in *Nanos2*-expressing XX gonads; and associated with the NANOS2 protein (Fig. 1a and Supplementary Table 1). One of the 16 genes was *Dazl*, which encodes an RNA-binding protein that promotes translation^23^,^24^,^25^ and is required for the expression of *Stra8* in XX germ cells in a specific genetic background^26^. Because *Nanos2*^−/−^ XY germ cells abnormally enter meiosis accompanied by upregulation of *Stra8* (ref. 20), opposite to the phenotypes seen in *Dazl*^−/−^ XX germ cells^26^, we speculated that *Dazl* could be a direct target of NANOS2 in preventing abnormal meiosis in XY germ cells.

To test whether NANOS2 represses *Dazl* expression in XY germ cells via a post-transcriptional mechanism, we examined the expression profile of *Dazl* in XY gonads by quantitative reverse transcription-PCR (RT-qPCR) using primer sets that distinguish the unspliced and spliced transcripts of *Dazl*. The expression level of *Dazl* was high at E13.5 but lower at E15.5 for both unspliced and spliced transcripts in *Nanos2*^+/−^ gonads (Fig. 1b), and was inversely correlated with the level of the NANOS2 protein^27^. Interestingly, however, the reduction in *Dazl* mRNA was prohibited, specifically that of the spliced transcripts, in *Nanos2*^−/−^ XY gonads (Fig. 1b). These data indicate that *Dazl* expression is post-transcriptionally repressed in XY germ cells in a *Nanos2*-dependent manner.

Since the Nanos complex binds to the 3′-untranslated region (UTR) of its target mRNAs in *Drosophila*^28^,^29^, we tested whether NANOS2 represses the level of the *Dazl* mRNA via its 3′-UTR. We generated a transgenic mouse line carrying a BAC for *Dazl* in which a 3 × FLAG tag was inserted at the carboxyl-terminus of DAZL and the *Dazl* 3′-UTR was flanked with FLP recombinase target (*Frt*) sequences (Fig. 1c). The FLAG-DAZL protein was specifically expressed in germ cells (Supplementary Fig. 2) and complemented the *Dazl*-deficient phenotype (Supplementary Fig. 3)^30^. Removal of the 3′-UTR, by crossing the *Dazl3Flag-Frt-3′-UTR-Frt-pA* transgenic mice (*Dazl3F*) with *Rosa-Flp* mice (to generate *Dazl3F;Flp* transgenic mice), increased *Flag-Dazl* mRNA expression in the transgenic XY gonads (Fig. 1d). As a consequence, the total amount of *Dazl* mRNA in the transgenic gonads reached that of *Nanos2*^−/−^ mice (Fig. 1d). The FLAG-DAZL protein level also increased twofold when the 3′-UTR was removed, which correlated with the increase of *Flag-Dazl* mRNA (Fig. 1d,e). These results suggest that the *Dazl* mRNA's 3′-UTR is required for its repression.

We next examined whether the repression of *Dazl* expression requires NANOS2. To this end, we compared *Flag-Dazl* expression in *Nanos2*^*+/+*^ and *Nanos2*^−/−^ backgrounds. We reasoned that if NANOS2 represses the expression of *Dazl* mRNA by associating with its 3′-UTR, the expression of *Flag-Dazl* mRNA should not be affected by the absence of the 3′-UTR in the *Nanos2*^−/−^ background, while the expression of *Flag-Dazl* carrying the 3′-UTR would increase in *Nanos2*^−/−^ gonads compared with *Nanos2*^*+/+*^ gonads. Indeed, removal of the 3′-UTR did not affect *Flag-Dazl* expression in *Nanos2*^−/−^ gonads, while the expression level increased in *Nanos2*^−/−^ when *Flag-Dazl* mRNA carries the 3′-UTR (Fig. 1f). Furthermore, RNA-immunoprecipitation (RIP) analysis revealed that the association between NANOS2 and *Flag-Dazl* decreased when the 3′-UTR was lacking (Fig. 1g). These data suggest that NANOS2 represses expression of the *Dazl* mRNA by associating with its 3′-UTR.

To further define the association between NANOS2 and *Dazl* 3′-UTR, we took advantage of our recent finding that NANOS2 requires DND1 to associate with its target RNAs^31^. Because NANOS2 localizes to processing bodies (P-bodies), cytoplasmic granules involved in RNA degradation and storage, in NIH/3T3 cells when DND1 was co-transfected as seen in XY germ cells^31^, we expected that association between NANOS2 and *Dazl* 3′-UTR may be recapitulated in the cultured cell by the presence of DND1. We performed RIP followed by RT-qPCR analysis using anti-FLAG antibody by transfecting FLAG-tagged NANOS2 and HA-tagged DND1 together with EGFP reporter carrying the *Dazl* 3′-UTR or *Oct4* 3′-UTR as a negative control. We found that FLAG-NANOS2 specifically associates with *Dazl* 3′-UTR in the presence of DND1 (Supplementary Fig. 4a). Importantly, amino acid substitution in zinc finger domain of NANOS2 (C61A and C96A), which is required for the binding to DND1 (ref. 31), abrogates the association. These data indicate that NANOS2 associates with *Dazl* 3′-UTR via interacting with DND1. We next divided the *Dazl* 3′-UTR into 3 fragments to narrow down the NANOS2-binding region (Supplementary Fig. 4b). RIP-qPCR analysis revealed that the interaction of FLAG-NANOS2 was enriched to the middle and 3′ fragments, suggesting the presence of selective binding in the 3′-UTR. Because these interactions were weaker than that in full-length 3′-UTR, it is likely that NANOS2 associates with multiple regions in the *Dazl* 3′-UTR. Although we successfully detected the interaction between NANOS2 and *Dazl* 3′-UTR in NIH/3T3 cell, the NANOS2-dependent suppression of GFP reporter was not observed, indicating that this system recapitulated target binding but not for RNA processing observed in germ cells.

### Excess DAZL prevents sexual differentiation of XY germ cells

**Figure 2:**
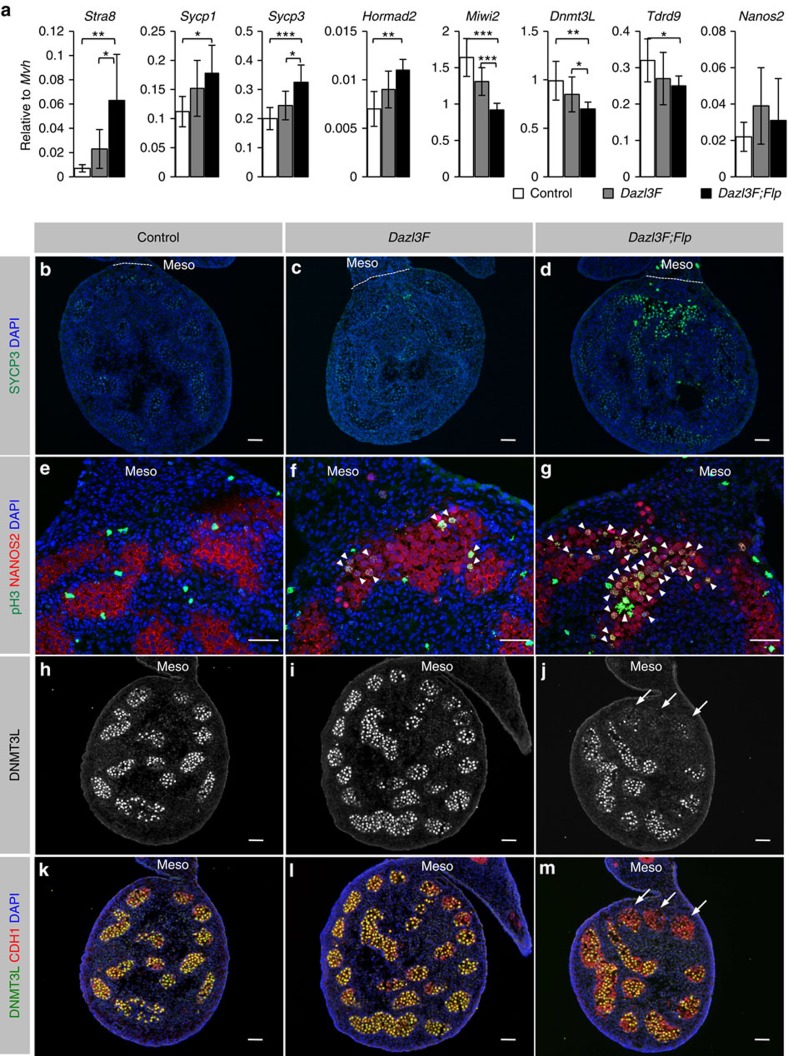
Excess DAZL abrogates sexual differentiation of XY germ cells. (**a**) RT-qPCR analysis of meiotic (*Stra8*, *Scyp1/3* and *Hormad2*) and male-specific (*Miwi2*, *Dnmt3L*, *Tdrd9* and *Nanos2*) genes in XY gonads at E15.5. The *y* axis shows the expression level relative to *Mvh*. Error bars, ±s.d. (*n*=3–8). Significance levels of changes are indicated (two-tailed Student's *t*-test; ***P<0.0005, **P<0.005, *P<0.05). (**b**–**m**) Immunostaining of XY germ cells at E15.5 with anti-SYCP3 (**b**–**d**), anti-pH3 (**e**–**g**) and anti-DNMT3L (**h**–**m**) antibodies. NANOS2 (**e**–**g**) and CDH1 (**k**–**m**) were used as germ cell markers. The mesonephros (meso) is at the top of each panel. DNA was counterstained with DAPI (blue). Arrowheads and arrows indicate pH3-positive germ cells and DNMT3L-negative tubules, respectively. Scale bar, 50 μm.

We next examined whether the repression of *Dazl* expression by NANOS2 is important for sexual differentiation of XY germ cells. RT-qPCR analysis showed that the expression of representative meiotic genes increased in the *Dazl3F;Flp* transgenic XY gonads in a 3′-UTR-dependent manner, whereas that of male-specific genes, except *Nanos2*, decreased in a 3′-UTR-dependent manner (Fig. 2a). Immunofluorescence (IF) analysis revealed that SYCP3, a component of the synaptonemal complex in meiotic cells, and pH3, a marker of mitotic cells, were abundant in the transgenic germ cells near the mesonephros when the 3′-UTR of the *Dazl* mRNA was removed (Fig. 2b–g). Conversely, in such germ cells, expression of a male-specific protein, DNMT3L, was suppressed (Fig. 2h–m). These expression patterns were similar to those in *Nanos2*^−/−^ XY germ cells^20^,^21^, suggesting that NANOS2-mediated repression of *Dazl* expression plays an important role in sexual differentiation of XY germ cells. However, the abnormalities induced by increased levels of DAZL were observed only in germ cells located near the mesonephros, unlike in *Nanos2*^−/−^ XY germ cells.

**Figure 3:**
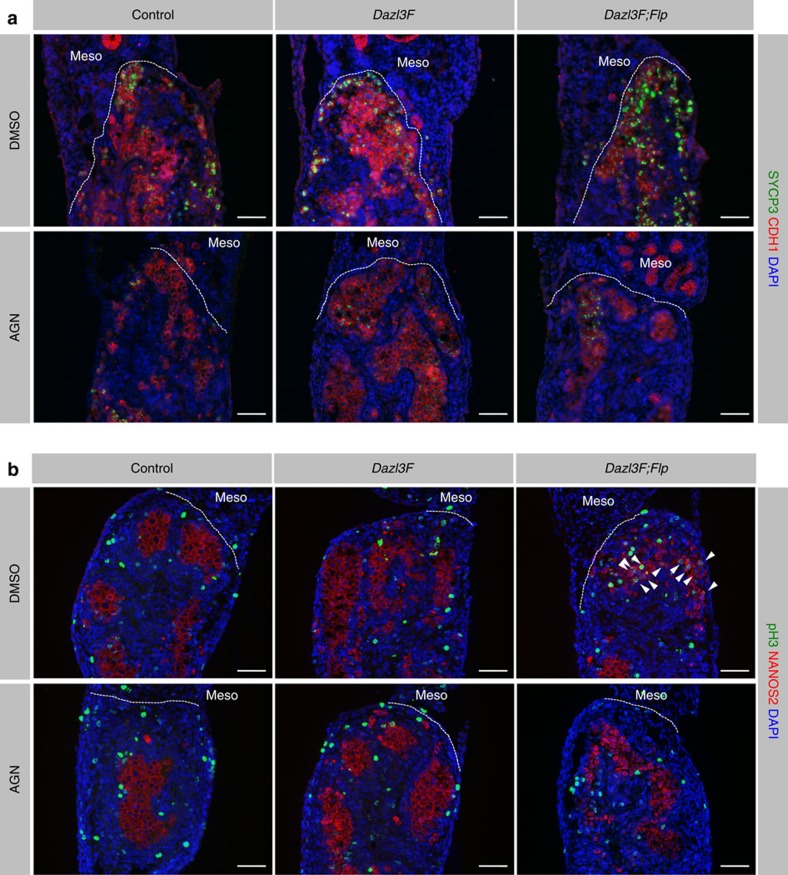
Dazl makes XY germ cells responsive to retinoic acid signalling. XY gonads harvested from E13.5 embryos were cultured with the RA receptor antagonist (AGN 193109) or DMSO vehicle for 48 h. (**a**,**b**) Immunofluorescence staining for (**a**) SYCP3 (green) and CDH1 (red), and for (**b**) pH3 (green) and NANOS2 (red). DNA was counterstained with DAPI. The mesonephros (meso) is at the top of each panel. Arrowheads indicate pH3-positive XY germ cells. Scale bars, 50 μm.

Although our *Dazl3F;Flp* transgenic mice did not fully recapitulate the *Nanos2*^−/−^ phenotype in all XY germ cells, the regional restriction observed suggests that the transgenic XY germ cells became responsive to extrinsic signal(s) from the mesonephros. Previous studies showed that RA influences the cell cycle state in XY germ cells^16^,^17^,^19^,^32^. Because it is believed that the mesonephros is a source of RA^16^, the regional restriction of the defects observed in the transgenic XY germ cells may be influenced by RA signalling. To test this possibility, we cultured *Dazl3F;Flp* transgenic XY gonads with or without an RA receptor antagonist, AGN 193109. IF analysis showed that the intense SYCP3 and pH3 signals observed in *Dazl3F;Flp* gonads were diminished when RA signalling was inhibited (Fig. 3a,b). These results suggest that excess DAZL makes XY germ cells responsive to RA signalling.

We next examined the consequences of the abrogated male-type differentiation in adult testes. We found that testes became smaller in the *Dazl3F;Flp* transgenic mice in a 3′-UTR-dependent manner (Supplementary Fig. 5). Moreover, the testicular weight was less in those transgenic mice with a *Nanos2*^+/−^ background but was recovered in a *Dazl*^+/−^ background (Supplementary Fig. 5a and b). Histological analysis revealed that seminiferous tubules showing defective spermatogenesis were increased in a 3′-UTR- and a *Nanos2*-dependent manner. Again, the defective spermatogenesis was recovered in a *Dazl*^+/−^ background (Supplementary Fig. 5c). These data suggest that NANOS2-dependent repression of *Dazl* expression is required for normal male germ cell development.

### Antagonistic interaction between NANOS2 and DAZL

NANOS2 interacts with a wide range of target RNAs aside from *Dazl* (Fig. 1a)^21^, implying that *Dazl* is not the sole target of NANOS2. However, removing the *Dazl* 3′-UTR alone resulted in defective male-type differentiation in *Dazl3F;Flp* transgenic XY germ cells. This raised the possibility of a functional interaction between NANOS2 and DAZL in XY germ cells. Since DAZL, as opposed to NANOS2, is implicated in activating translation of its target RNAs, we hypothesized that NANOS2 could counteract this activity of the DAZL protein in order to regulate other target RNAs, in addition to the repression of *Dazl* expression described above.

**Figure 4:**
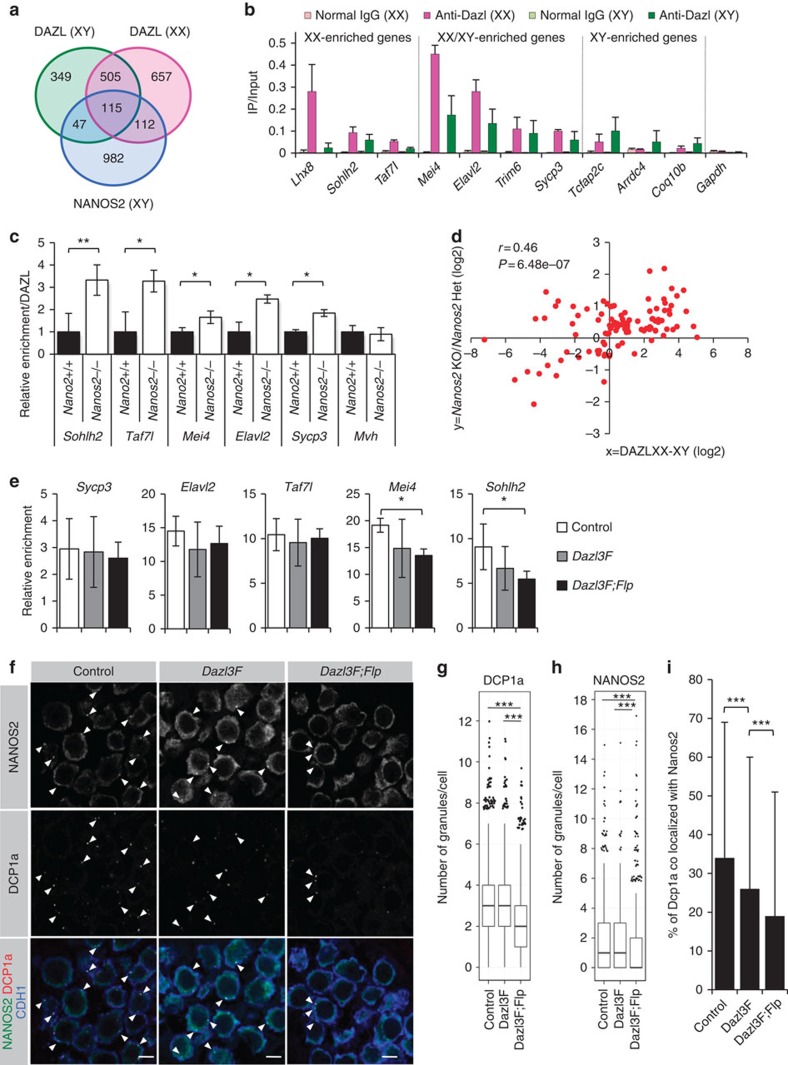
Antagonistic interaction between NANOS2 and DAZL in XY germ cells. (**a**) Venn diagrams show the overlap among DAZL-associated gene probes in XX (magenta) and XY gonads (green), and NANOS2-associated probes in XY gonads (blue) at E14.5. Probes showing fourfold higher levels (*P*<0.05) in the IP than in the input were selected. (**b**) RIP and RT-qPCR analysis of selected DAZL-associated genes. RIP was performed with an anti-DAZL antibody. The *y* axis represents the relative enrichment of IP to input. Error bars, ±s.d. (*n*=3). (**c**) RIP performed with anti-DAZL antibody using *Nanos2*^*+/+*^and *Nanos2*^−/−^ XY gonads at E14.5. The relative enrichment of selected DAZL-associated mRNAs in IPs was measured by qPCR. To compare the amounts of the precipitated mRNAs in the *Nanos2*^*+/+*^ and *Nanos2*^−/−^ gonads, sample volumes for cDNA synthesis were normalized to the immunoprecipitated DAZL protein. Error bars, ±s.d. (*n*=3). Significance levels of changes are indicated (Mann–Whitney *U*-test; **P<0.005, *P<0.05). (**d**) Correlation analysis between the association of DAZL and the expression changes of the DAZL and NANOS2-associated genes in *Nanos2*^−/−^ XY gonads. The *x* axis represents the difference in IP/input of DAZL in XX versus XY gonads. The *y* axis represents relative expression changes of the genes in *Nanos2*^−/−^ versus *Nanos2*^*+/+*^ gonads. Red dots indicate 274 overlapping probes in **a** showing statistical reliability (two-tailed Student's *t*-test; *P*<0.05) in our previous microarray analyses^21^. *r*, correlation efficient. (**e**) RNA-immunoprecipitation and RT-qPCR analysis, using anti-NANOS2 antibody, of XY gonads at E15.5 as same with Fig. 1g. (**f**) Immunostaining of XY germ cells at E15.5. CDH1 was used as a germ cell marker. Arrowheads indicate granules where NANOS2 and DCP1a are co-localized. Scale bars, 5 μm. (**g**,**h**) DCP1a (**g**) and NANOS2 (**h**) granules in XY germ cells quantified using single planes of confocal photomicrographs. Horizontal bars indicate the mean values. (**i**) The proportion of DCP1a granules co-localized with NANOS2 granules. (**g**–**i**) The significance of changes is indicated (Steel–Dwass all-pairs non-parametric test; ***P<0.0005). IP, immunoprecipitate.

To test this possibility, we examined whether NANOS2 and DAZL interact with common RNA species. Based on RIP followed by microarray analysis, we identified DAZL-associated mRNAs in XX and XY gonads at E14.5 (Fig. 4a and Supplementary Fig. 6). We compared these data with the NANOS2-associated mRNA data, and found that 21.8% (274/1256) of NANOS2-associated probes also bound DAZL. The commonly associated mRNAs included those of genes involved in meiosis and oogenesis, and of *Dazl* itself (Fig. 4a and Supplementary Data 1). The microarray results were supported by RT-qPCR (Fig. 4b). Notably, the associations between DAZL and the 112 probes enriched in XX gonads were diminished in XY gonads (Fig. 4a). To test if the sex-biased DAZL association is due to NANOS2, we examined the association between DAZL and the mRNAs in *Nanos2*-deficient XY germ cells. We found that the mRNAs that were more strongly associated with DAZL in XX gonads than XY gonads were more abundant in the immunoprecipitated DAZL fraction from *Nanos2*^−/−^ XY gonads (Fig. 4c). Furthermore, the difference in the association between DAZL and mRNAs in XX and XY gonads was positively correlated with increased expression of the cognate genes in *Nanos2*^−/−^ XY gonads (*r*=0.46, *P*=6.48e−7) (Fig. 4d). These results suggest that NANOS2 antagonizes DAZL through preventing its binding to common target mRNAs in XY germ cells.

Next we asked whether excess DAZL impairs NANOS2 function in XY germ cells. To this end, we first examined the influence of excess DAZL on the binding of NANOS2 to the NANOS2-associated mRNAs in transgenic XY germ cells. The results of RIP-qPCR analysis showed that the binding of NANOS2 to two out of five target mRNAs was decreased in a 3′-UTR-dependent manner (Fig. 4e), suggesting that DAZL prevents the binding of NANOS2 to some common targets. To examine this scenario, we performed RIP-qPCR analysis by immunoprecipitating FLAG-DAZL protein. The results showed that the association of FLAG-DAZL was more abundant in four out of the five target mRNAs in *Dazl3F;Flp* than *Dazl3F* transgenic XY germ cells (Supplementary Fig. 7a). Furthermore, the observed stronger association was correlated with the increased expression of cognate genes in a 3′-UTR-dependent manner (Supplementary Fig. 7b). These data suggest that excess DAZL impairs NANOS2 function via preventing its binding of some target RNAs.

We next examined the localization of NANOS2 to P-bodies. Because the number of P-bodies is decreased in *Nanos2*^−/−^ XY germ cells, it is supposed that NANOS2 is involved in P-body assembly, which also affects RNA metabolism in XY germ cells^12^. We reasoned that DAZL might also affect NANOS2 localization to P-bodies because of reduction of RNA binding to NANOS2. To examine the influence of excess DAZL on P-body formation in transgenic XY germ cells, we performed IF that was followed by quantitative image analyses (Supplementary Fig. 8). We found that the number of granules expressing DCP1a, a P-body component, was reduced in *Dazl3F;Flp* transgenic XY germ cells where the *Dazl* 3′-UTR was removed (Fig. 4f,g), again recapitulating the *Nanos2*^−/−^ phenotype^12^. Interestingly, the number of NANOS2 granules was also reduced in the transgenic XY germ cells in a 3′-UTR-dependent manner (Fig. 4f,h). Furthermore, the fraction of DCP1a that co-localized with NANOS2 was decreased in the transgenic germ cells in a 3′-UTR-dependent manner (Fig. 4i). In contrast, DAZL protein was distributed in the cytoplasm but its co-localization to P-bodies was less than that of NANOS2 (Supplementary Fig. 9). These results suggest that excess DAZL impairs localization of NANOS2 to P-bodies, which leads to the reduction of degradation rate of Nanos2-associated RNAs. This may also account for one of suppression mechanisms of NANOS2 function by DAZL.

## Discussion

In this study, we showed that NANOS2 post-transcriptionally represses *Dazl* mRNA expression in XY germ cells. Intriguingly, lack of the *Dazl* 3′-UTR alone phenocopied *Nanos2*-deficient phenotypes, although the defects of *Nanos2*-deficiency were not fully recapitulated in our *Dazl3F;Flp* transgenic mice. Because we also found that NANOS2 has an antagonistic effect on DAZL, the regional restriction of the defects may be due to the presence of NANOS2 in the transgenic XY germ cells. However, this milder phenotype led us show that the transgenic XY germ cells became susceptible to RA. Our data suggest that *Dazl* is a crucial target of NANOS2 to promote sexual differentiation of XY germ cells, and at the same time provide the first *in vivo* evidence of the identification of a mammalian NANOS target.

**Figure 5:**
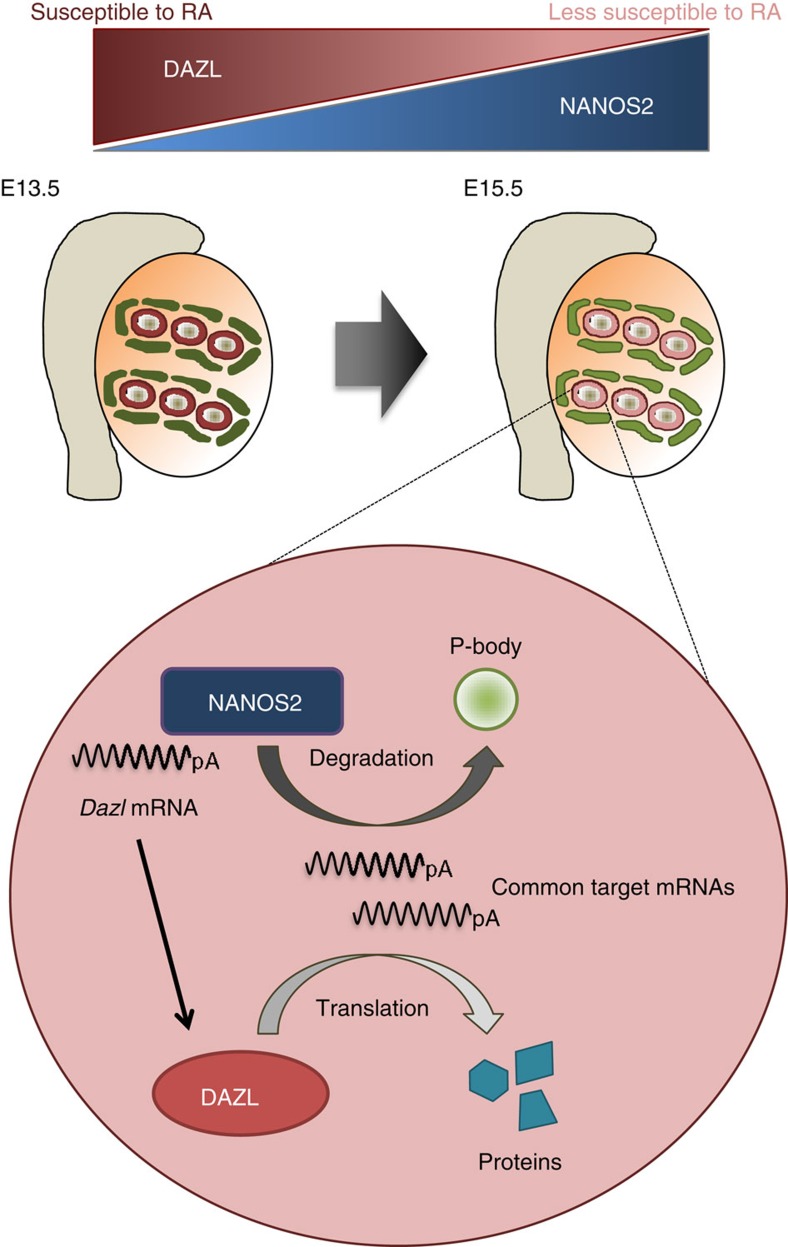
Working model of sexual differentiation of XY germ cells in the mouse. At the beginning of sexual differentiation, *Dazl* is strongly expressed in XY germ cells. While the stronger *Dazl* expression makes XY germ cells susceptible to RA signalling, meiosis is prohibited by strong expression of *Cyp26b1* in sertoli cells (dark green). However, *Cyp26b1* expression is decreased after E14.5 in the XY gonad (light green), suggesting that this anti-proliferative/meiotic mechanism becomes weaker. In contrast to the reduction of *Cyp26b1* expression, *Nanos2* expression becomes stronger in XY germ cells. A major function of NANOS2 is safeguarding XY germ cells from RA by reducing the level of the *Dazl* mRNA and antagonizing the DAZL protein.

In mice, PGCs acquire competence to enter meiosis after they reach embryonic gonads, regardless of gonadal sex^33^. It is likely that DAZL is involved in this process, because *Dazl*-deficient XX PGCs were unable to enter meiosis in a specific genetic background^26^. Whereas *Dazl* is strongly expressed at the beginning of sexual differentiation of XY germ cells (Fig. 1b), meiosis is suppressed in embryonic XY germ cells due to the function of CYP26b1 in Sertoli cells^19^. However, even in XY gonads, germ cells may be exposed to RA, which facilitates resumption of the cell cycle^32^, due to the reduction of *Cyp26b1* expression after E14.5 (ref. 16). We showed that a molecular mechanism involved in the maintenance of mitotic quiescence is *Dazl* suppression via NANOS2. Because excess DAZL makes XY germ cells responsive to RA signalling, our data suggest that NANOS2-mediated *Dazl* suppression acts to safeguard XY germ cells from RA signalling (Fig. 5). How DAZL makes XY germ cells susceptible to RA is an interesting question. Since DAZL is required for *Stra8* expression at the onset of the sexual differentiation of XX germ cells^26^, DAZL may cause XY germ cells to become responsive to RA signalling by the same mechanism as in XX germ cells.

By taking advantage of a BAC transgenic system, we showed that NANOS2 represses expression of the *Dazl* mRNA via association with its 3′-UTR in sexually differentiating XY germ cells, indicating the usefulness of this system for investigating the effect of the 3′-UTR *in vivo*. How NANOS2 associates with the *Dazl* mRNA remains an open question. We recently reported that NANOS2 requires a germ cell-specific RNA-binding protein, DND1, to exert its function in XY germ cells^31^. Based on this knowledge we succeeded to reproduce NANOS2 binding to *Dazl* mRNA in a cultured cell line, although the suppression of gene expression was not achieved. We also noticed that NANOS2 might associate with not only the 3′-UTR but also other regulatory elements, because removing *Dazl* 3′-UTR resulted in milder reduction of the binding of *Dazl* mRNA to NANOS2 (Fig. 1g). Although further analysis is required to understand the mechanism of NANOS2-mediated gene regulation, identification of a target RNA *in vivo* provides a reliable test element for future studies.

Intriguingly, the identified NANOS2 target, the *Dazl* mRNA, also encodes an RNA-binding protein. Because DAZL, as opposed to NANOS2, is implicated in activation of translation, it is likely that post-transcriptional regulation of functionally opposing RNA-binding proteins plays a pivotal role in sexual differentiation of XY germ cells in mice. In this regard, it is possible that DAZL counteracts the effect of NANOS2, because excess DAZL impaired the binding of NANOS2 to some common targets (Fig. 4e) and NANOS2 granule formation coinciding with the reduction of P-bodies in *Dazl3F;Flp* transgenic XY germ cells (Fig. 4e–i). This mutual antagonism between NANOS2 and DAZL might be a mechanism that controls the cellular state of XY germ cells (Fig. 5). How these RNA-binding proteins determine the fate of their target RNA remains an open question. A simple explanation might be that NANOS2 and DAZL competitively bind to target RNAs, as reported for the Oskar and Smaug proteins in the regulation of the *Nanos* RNA in early development of *Drosophila*^34^. Because the binding of DAZL to several RNAs was stronger in *Nanos2*^−/−^ XY germ cells (Fig. 4c), NANOS2 might disturb the interaction between DAZL and its target RNAs. However, it is also possible that these two RNA-binding proteins separately bind to the same RNAs because the binding of NANOS2 was not fully influenced by excess DAZL (Fig. 4e). It is likely that the mode of interaction and antagonism depends on the sequence of each mRNA. In either case, the number of NANOS2 and/or DAZL molecules interacting with a target RNA must contribute to the decision on its fate. Although it was shown that DAZL recognizes the simple sequence, GUU^35^, via a structural studies, *in vivo* binding studies indicate that the rule cannot be applied to all target RNAs^24^,^36^. Therefore, the binding specificity of RNA-binding proteins would be brought by several mechanisms including the sequence flanking to the core motif and other RNA-binding proteins. To address this issue, detailed biochemical analyses using *in vivo* samples are required.

In summary, we have provided *in vivo* evidence that the *Dazl* mRNA is a direct target of NANOS2, and a molecular basis for how NANOS2 regulates the male differentiation pathway. Because *Nanos2* and *Dazl* are conserved in mammals, our findings may also contribute to a better understanding of the sexual differentiation of XY germ cells in humans. Recent studies have demonstrated that PGC-like cells are induced from murine embryonic stem cells and induced pluripotent stem cells^37^,^38^, prompting efforts to produce functional gametes *in vitro*. We believe that our data may also contribute to basic research towards *in vitro* production of sexually differentiated male germ cells.

## Methods

### Mice

Mice were housed in a specific-pathogen-free animal care facility at the National Institute of Genetics (NIG). All experiments were approved by the NIG Institutional Animal Care and Use Committee. For induction of *3* × *Flag-Nanos2* in XX PGCs, tamoxifen (3 mg) was administered to pregnant female mice at E10.5. Gene-targeted mice (*Nanos2*^−/−^, *Dazl*^−/−^ and *Stra8-GFP* knock-in) and transgenic mouse lines (*CAG-CAT*^*flox*^*-3FlagNanos2-pA* and *Oct4-CreER*^*T2*^*-pA*), were as described^10^,^20^,^21^,^22^,^30^. *Rosa-Flp* mice were provided by S.M. Dymecki^39^. All knock-in and knockout mouse lines used in this study were in mixed genetic background (between C57BL/6 and ICR). A BAC-expressing transgenic mouse line, *Dazl3Flag-Frt-3′-UTR-Frt-pA*, was generated by two-step BAC recombination as follows. A BAC clone (PR-23-308G11, Invitrogen) that carries the entire *Dazl* sequence and a partial sequence encoding *Rftn1* was used for recombination. First, a recombination cassette, *3Flag-stop-Frt-Kanamycin-Frt*, was electroporated into competent cells expressing λred recombinase and was introduced at the end of the *Dazl* coding DNA sequence in frame. After removing *Kanamycin* by transforming the cells with a plasmid expressing the Flp recombinase (pCP20), a second recombination cassette, *Kanamycin-Frt-rabbit β-globin poly(A)*, was introduced 540 bp downstream of the *Dazl* 3′-UTR. DNA purified from the resulting recombinant BAC clone was digested with restriction enzymes to remove vector sequences, and purified by gel elution. BAC-bearing transgenic mice were generated by microinjecting DNA into fertilized eggs, which were then transferred into the oviducts of pseudo-pregnant female mice. Five transgenic mice were produced, but three did not transmit the transgene to their offspring. Two mice did transmit the transgene to their offspring, and one line used in this study stably expressed *Flag-Dazl* in the gonads for more than six generations. The BAC transgenic mice were in mixed genetic background (between C57BL/6, C3H/HeN and ICR).

### RT-qPCR

Total RNA was extracted from embryonic gonads using RNeasy Mini Kits (Qiagen). Aliquots (100 ng) of total RNA were incubated with SuperScript III reverse transcriptase (Invitrogen) for 60 min at 50 °C to synthesize cDNA. Oligo (dT) (500 ng) was used for cDNA synthesis, except for the analysis of spliced and unspliced transcripts in Fig. 1b, for which a random 6-mer sequence (1 μg) was used. Quantitative PCR was carried out using KAPA SYBR Fast qPCR Kits (Nippon Genetics, Tokyo, Japan) and a Thermal Cycler, Dice Real Time System Single (Takara, Shiga, Japan). In each experiment, the PCR was replicated three times. Ct values were calculated by the second derivative maximum method and relative quantities of each mRNA were calculated using the ΔΔC_T_ method. We chose murine *vasa* homologue (*Mvh;* also known as *Ddx4*) as a normalizer based on the fact that its mRNA level is constant in wild type, *Nanos2*^−/−^ and BAC transgenic male gonads. Primers are listed in Supplementary Table 2.

### Western blotting

Western blotting (WB) was performed using a standard protocol. Whole-embryonic gonads or pieces of adult testis were lysed, and the proteins were separated in SDS–polyacrylamide gels and transferred to nitrocellulose or polyvinylidene fluoride membranes. To detect FLAG, DAZL, MVH and β-TUBULIN, the respective anti-FLAG M2 (1:10,000, Sigma-Aldrich, F3156), anti-DAZL (1:1,000, Abcam, ab34139), anti-MVH (1:1,000, Abcam, ab13840) or anti-β-TUBULIN (1:5,000, Sigma-Aldrich, 1A6) antibodies were reacted with membranes for 1 h at room temperature. After washing the membranes, the secondary antibodies, horse/goat anti-mouse/rabbit immunoglobulin G (IgG) conjugated with horseradish peroxidase (1:2,000, Cell Signaling, #7076S and #7074) was reacted with membranes for 30 min at room temperature. Signals were detected using SuperSignal West Femto Chemiluminescent Substrate Kits (Thermo Scientific). Images were acquired using the Ez-Capture MG chemiluminescence imaging system (Atto, Tokyo, Japan). All uncropped WB can be found as in the Supplementary Fig. 10. The FLAG-DAZL protein signals were quantified using ImageJ software.

### Histology

Testes from 10-week-old mice were fixed with 4% paraformaldehyde overnight at 4 °C. After being washed in PBS, testes were dehydrated and embedded in paraffin wax following a standard protocol. Blocks were sectioned at a width of 6 μm and the sections applied to glass slides. Haematoxylin and eosin staining was carried out following a standard protocol.

### Immunofluorescence staining

Embryonic gonads were fixed in 4% paraformaldehyde for 0.5–1 h at 4 °C and infiltrated with 10 or 20% sucrose in PBS for 0.5–1 h at 4 °C. Gonads were then embedded in Tissue-Tek O.C.T. compound (Sakura Finetek, Tokyo, Japan), and frozen in liquid nitrogen. Each gonad was transversely cryosectioned at 8 μm, and every seventh and ninth section, to cover the whole gonad, were applied to glass slides. Sections were reacted with primary antibodies overnight at 4 °C at the following dilutions: anti-NANOS2 (1:100, gift from Atsushi Suzuki, Yokohama National University), anti-SYCP3 (1:500, gift from Shinichro Chuma, Kyoto University, and 1:400, Abcam, ab97672), anti-pH3 (1:20, gift from Hiroshi Kimura, Tokyo Institute of Technology)^40^, anti-DNMT3L (1:500, gift from Shinya Yamanaka, iCeMS, Kyoto University), anti-TRA98 (1:2,000, gift from Yoshitake Nishimune, Osaka University), anti-FLAGM2 (1:10,000, Sigma-Aldrich, F3156), anti-DAZL (1:200, Abcam, ab34139), anti-DMC1 (1:200, Santa Cruz Biotechnology, sc-8973), anti-DCP1a (1:200, Abnova, Taipei, Taiwan, H00055802-M06), anti-CDH1 (1:400, R&D Systems, AF748) and anti-green fluorescent protein (GFP) (1:500, Abcam, ab13970). Secondary antibodies labelled with Alexa Fluor 488 and 594 (1:1,000, Molecular Probes), and Cy5 (1:1,000, Millipore) were used. DNA was counterstained with DAPI (100 ng ml^−1^). Fluorescence micrographs were acquired using an Olympus BX61 or FV1200 microscope, and were processed with the MetaMorph (version 7.0, Molecular Devices, Sunnyvale, CA, USA) or FV10-ASW (version 4.0) software packages.

### RNA-immunoprecipitation

For RIP and RT-qPCR analysis, lots of 10 gonads harvested from E14.5 or 15.5 embryos were homogenized in IP buffer (20 mM HEPES/KOH, pH7.5, 150 mM NaCl, 2.5 mM MgCl_2_, 0.1% NP-40, 1 mM dithiothreitol, 1 × protease inhibitor cocktail, 100 U ml^−1^ RNase inhibitor). After removing debris by centrifuging the lysates at 10,000*g* at 4 °C, supernatants were incubated with magnetic beads conjugated with protein G (Invitrogen, 10003D), which was pre-incubated with normal rabbit IgG (1:10, Santa Cruz Biotechnology, sc-2027), or anti-NANOS2 (1:40, as above) or anti-DAZL (1:200, Abcam, ab34139) or anti-FLAG M2 (1:200, Sigma-Aldrich, F3156) antibodies, for 2 h, washed with IP buffer, then incubated for 6 h at 4 °C with rotation. For microarray analysis, cellular extracts obtained from lots of 50 gonads were incubated with anti-DAZL antibody (1:50, Abcam, ab34139). Bead–antibody complexes were washed three times with IP buffer and transferred to new 1.5 ml tubes. After removing the IP buffer, precipitated RNAs and proteins were eluted by incubating the bead–antibody complexes with elution buffer (IP buffer containing 0.5% SDS) for 5 min at 37 °C. Input and unbound fractions (5% of starting materials) were taken from the centrifuged supernatants or the supernatants of IP reactions on a magnetic stand, respectively. Input and precipitated RNAs were dissolved in TRIzol reagent (Invitrogen) and isolated according to the manufacturer's protocol. To normalize the immunoprecipitated DAZL or FLAG-DAZL protein, the DAZL protein signals in WB were quantified using ImageJ software.

### Cultured cell experiments

FLAG-NANOS2**-**expressing vector was generated by inserting *Nanos2*-coding sequence into p3xFLAG-CMV7.1 vector (Sigma-Aldrich), and HA-DND1 expressing vector was generated by inserting HA-tagged Dnd1 coding sequence into pCDNA3.1 vector (Addgene). EGFP reporters were generated by inserting *Dazl* or *Oct4* 3′-UTR sequences into pEGFP-C2 vector (Clontech). One microgram of FLAG-NANOS2 and HA-DND1 expressing vectors together with same amount of EGFP reporters was transfected into 2 × 10^5^ NIH/3T3 (ATCC) cells by Lipofectamine 2000 (Thermo Fisher Scientific) according to the manufacturer's protocol. Twenty-four hours after transfection, cells were dissociated by 0.15% trypsin and were collected by centrifugation (1,000 r.p.m. for 2 min at 4 °C). RIP was carried out by using anti-FLAG M2 (1:200, as above) antibody as described above.

### Microarray analysis

Microarray analyses were carried out as described previously^20^,^21^. To identify DAZL-associating mRNAs, input and immunoprecipitated RNAs were isolated as described above. RNA quality was checked using a 2100 Bioanalyzer (Agilent). RNAs were reverse-transcribed and labelled with Cy3 using Low RNA Input Linear Amplification Kits (Agilent). The Cy3-labelled complementary RNAs were hybridized to a Whole Mouse Genome Oligo Microarray (G4122F, Agilent) using Gene Expression Hybridization Kits (Agilent) according to the manufacturer's procedure. A Microarray Scanner System (G2565BA, Agilent) was used for scanning arrays, and the generated images were processed using Feature Extraction software (version 9.1, Agilent). The data obtained from two replicates for each sample were processed using the Subio Platform (version 1.16, Subio, Kagoshima, Japan) as follows: all values <1 were replaced with 1, the data were normalized to the 75th percentiles, and fold-changes were calculated from log_2_-transformed signal intensities.

### Organ culture

Organ culture experiments were carried out as described^41^. XY gonads with the mesonephros were harvested from E13.5 embryos and cultured on the surface of culture membranes floating on DMEM with 10% horse serum, 100 units per ml penicillin and 100 units per ml streptomycin. In each experiment, pairs of XY gonads were cultured separately with DMSO or the RA receptor antagonist, AGN 193109 (5 μM, Toronto Research Chemicals, Toronto, ON, Canada). Cultures were maintained at 37 °C in 5% CO_2_ in humidified air for 48 h.

### Co-localization analysis

To quantify P-body-like structures, gonadal sections were subjected to triple-label immunofluorescence for CDH1, DCP1a, and NANOS2 or DAZL. Confocal images were taken with a Zeiss LSM510 scanning confocal microscope with a × 100 oil-immersion objective lens, at a 1,024 × 1,024 pixel resolution and 1 × zoom setting. Identical gain, offset, and acousto-optic tunable filter settings were used for all samples prepared on the same day. Captured images were analysed automatically using a custom program written for the R software environment (www.r-project.org) with the EBImage and RImageBook packages^42^. Image segmentation of individual germ cells was performed based on CDH1 staining of germ cell membranes. In brief, germ cell masks were created by applying a Gaussian blur filter followed by local thresholding, skeletonization, hole filling and filtering with the SD of the object's radius. Granular objects immunopositive for NANOS2 or DAZL and DCP1a were detected by applying the difference of Gaussian filters followed by thresholding and size selection. The numbers of granular objects in individual germ cells were counted and pooled for statistical analyses. Box plots were obtained from 1,081 (control), 857 (Dazl3F) and 1,181 (Dazl3F;Flp) germ cells.

### Software availability

The R scripts for the quantification of NANOS2 and DCP1a granules is available on the following website https://github.com/tkatsuki/NatureComm2016

## Additional information

**Accession codes:** Microarray data have been deposited in the Gene Expression Omnibus (GEO) database under accession code GSE56795.

**How to cite this article**: Kato, Y. *et al*. *Dazl* is a target RNA suppressed by mammalian NANOS2 in sexually differentiating male germ cells. *Nat. Commun.* 7:11272 doi: 10.1038/ncomms11272 (2016).

## Supplementary Material

Supplementary Information Supplementary Figures 1-10 and Supplementary Tables 1-2

Supplementary Data This file contains a data set of RNAs assocaiting with NANOS2 and DAZL
