# Supplementary material for: Dazl is a target RNA suppressed by mammalian NANOS2 in sexually differentiating male germ cells
Source: Nat Commun. 2016 Apr 13;7:11272. doi: 10.1038/ncomms11272 (PMC4833867; doi:10.1038/ncomms11272)
Supplement: Supplementary Information — Supplementary Figures 1-10 and Supplementary Tables 1-2 [file ncomms11272-s1.pdf]

Supplementary Figure 1

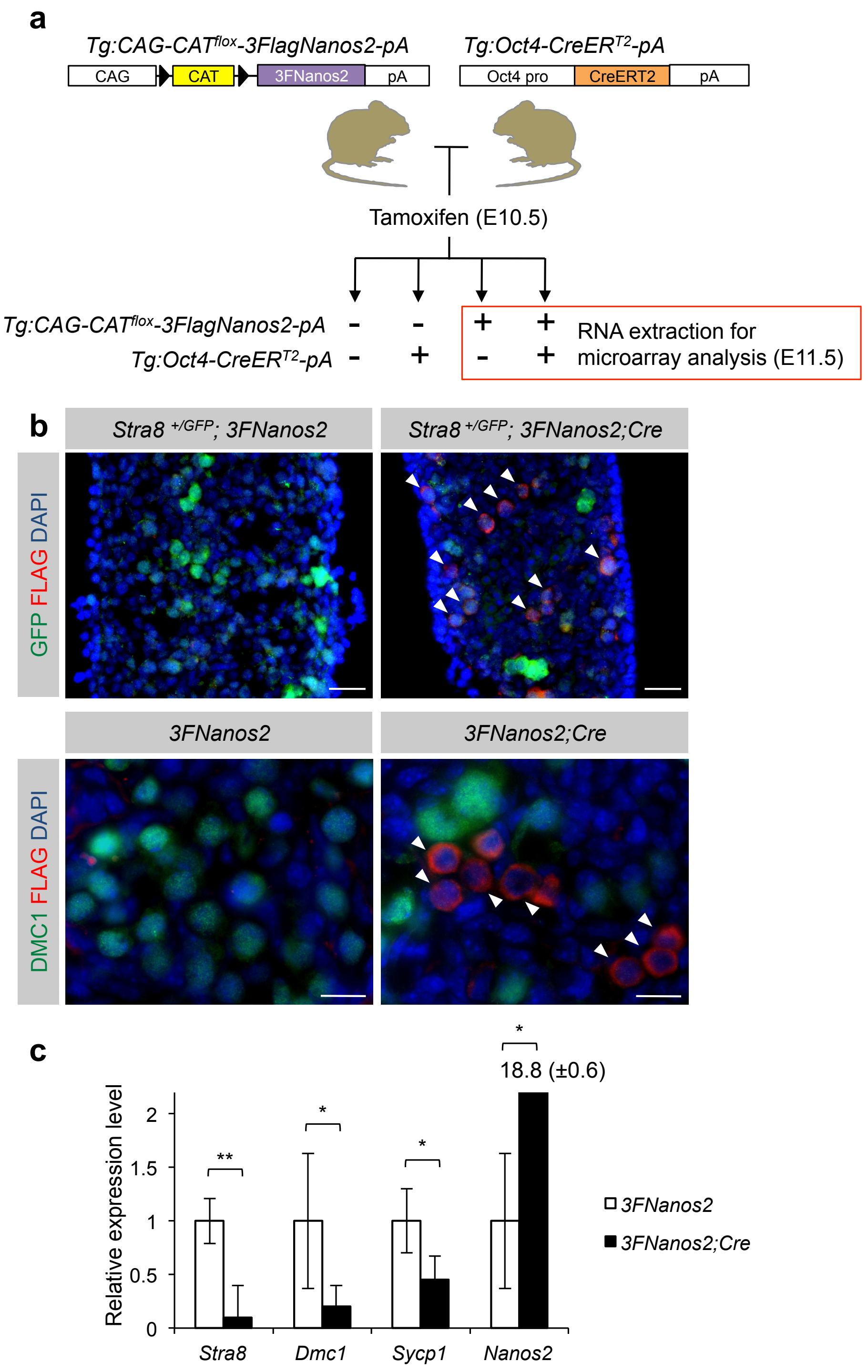

Supplementary Fig. 1. Ectopic induction of FLAG-tagged NANOS2 in XX primordial germ cells (**a**) Schematic drawing of the strategy used to induce FLAG-tagged NANOS2 expression in XX PGCs. A transgenic (Tg) mouse line, *CAG-CAT*^{flox}*-3FlagNanos2-pA* was crossed with a Cre-expressing mouse line, *Oct4-CreER*^{T2}*-pA*. Tamoxifen (3 mg) was administered to pregnant female mice at E10.5, and total RNAs were isolated from XX gonads at E11.5 for microarray analysis. (**b** and **c**) FLAG-tagged NANOS2 suppresses meiosis in XX PGCs. (**b**) Immunofluorescence staining of STRA8 and DMC1 in transgenic XX gonads at E12.5 (upper panels, scale bar = 50 μm) and E14.5 (lower panels, scale bar = 20 μm). STRA8 expression was examined by using *GFP* gene knock-in *Stra8* heterozygous mice[5] and was detected using an anti-GFP (green fluorescent protein) antibody. Arrowheads indicate FLAG-NANOS2-positive XX PGCs. (**c**) Quantitative reverse transcription-PCR (RT-qPCR) analysis of meiotic genes in the *Nanos2*-expressing XX gonads at E12.5. The y-axis shows the expression levels of meiotic genes in *Nanos2*-expressing XX gonads (black bars), relative to controls (white bars). The expression level of each gene was normalised against the murine *vasa* homolog. Error bars, ±SD (n = 4).

Supplementary Figure 2

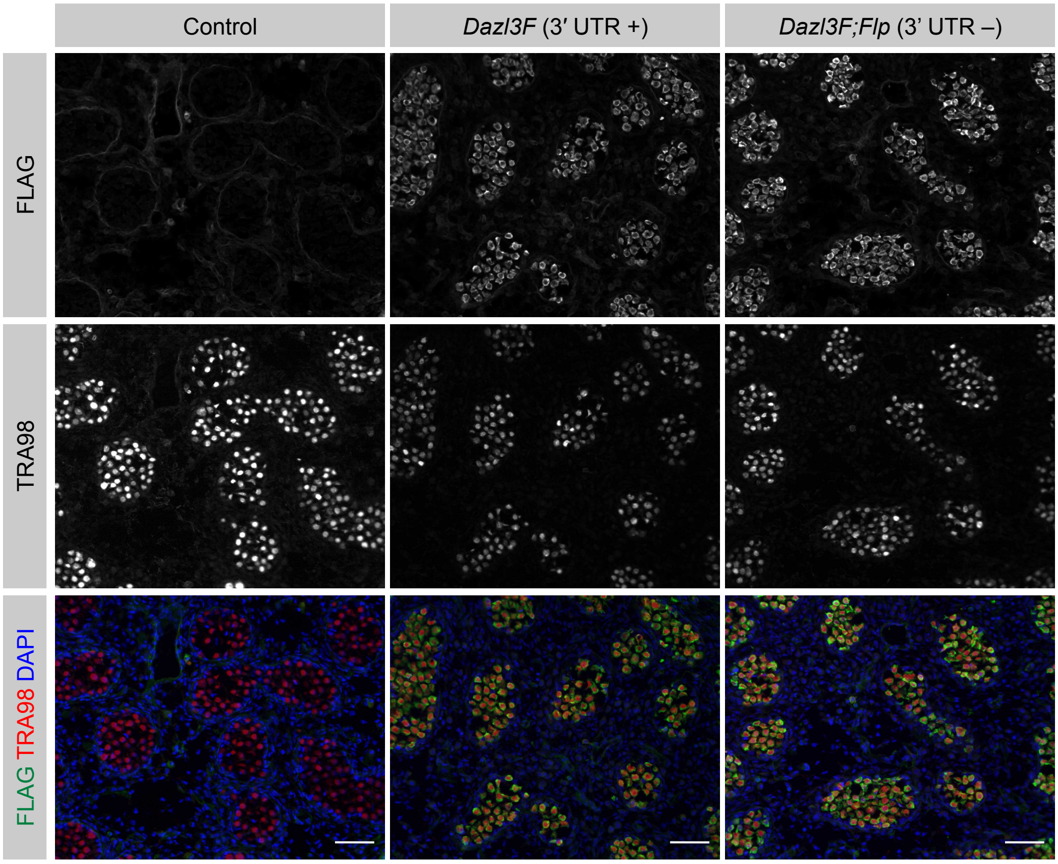

Supplementary Fig. 2. Germ cell-specific expression of FLAG-DAZL The spatial expression pattern of FLAG-DAZL protein was examined by immunostaining.

Transgenic XY gonads at E15.5 were stained with anti-FLAG and a germ cell marker, anti-TRA98, antibodies. DNA was counterstained with DAPI. Scale bars = 50 μm.

Supplementary Figure 3

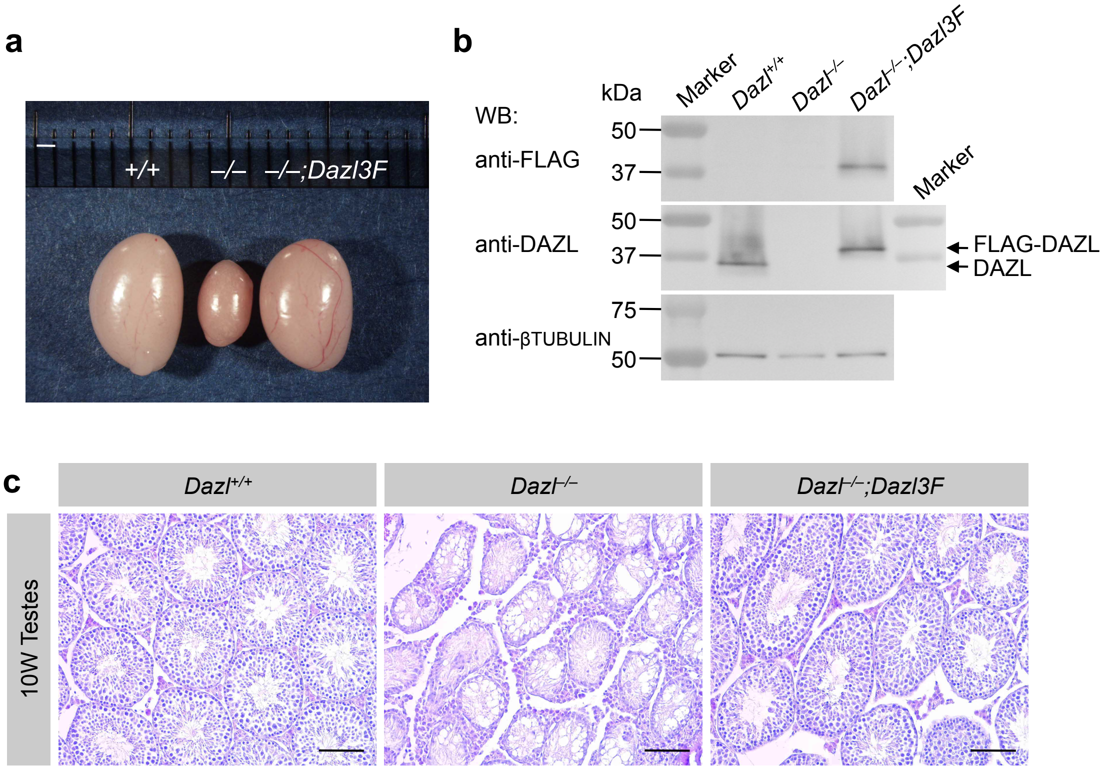

Supplementary Fig. 3. BAC-expressing transgenic mice rescued the defective spermatogenesis in *Dazl* mutants (**a**) A photograph shows 10-week-old (10W) testes

of *Dazl*$^{+/+}$ (+/+), *Dazl*$^{-/-}$ (–/–), and *Dazl*$^{-/-}$*;Dazl3F* (–/–;Dazl3F) mice. Scale bar = 1 mm. (**b**) Genotypes of all mice in panel **a** were confirmed by western blotting (WB). (**c**) Hematoxylin and eosin staining of 10W testes of each genotype. Note that all types of spermatogenic cells were observed in the *Dazl*$^{-/-}$*;Dazl3F* testes. Scale bars = 100 μm.

Supplementary Figure 4

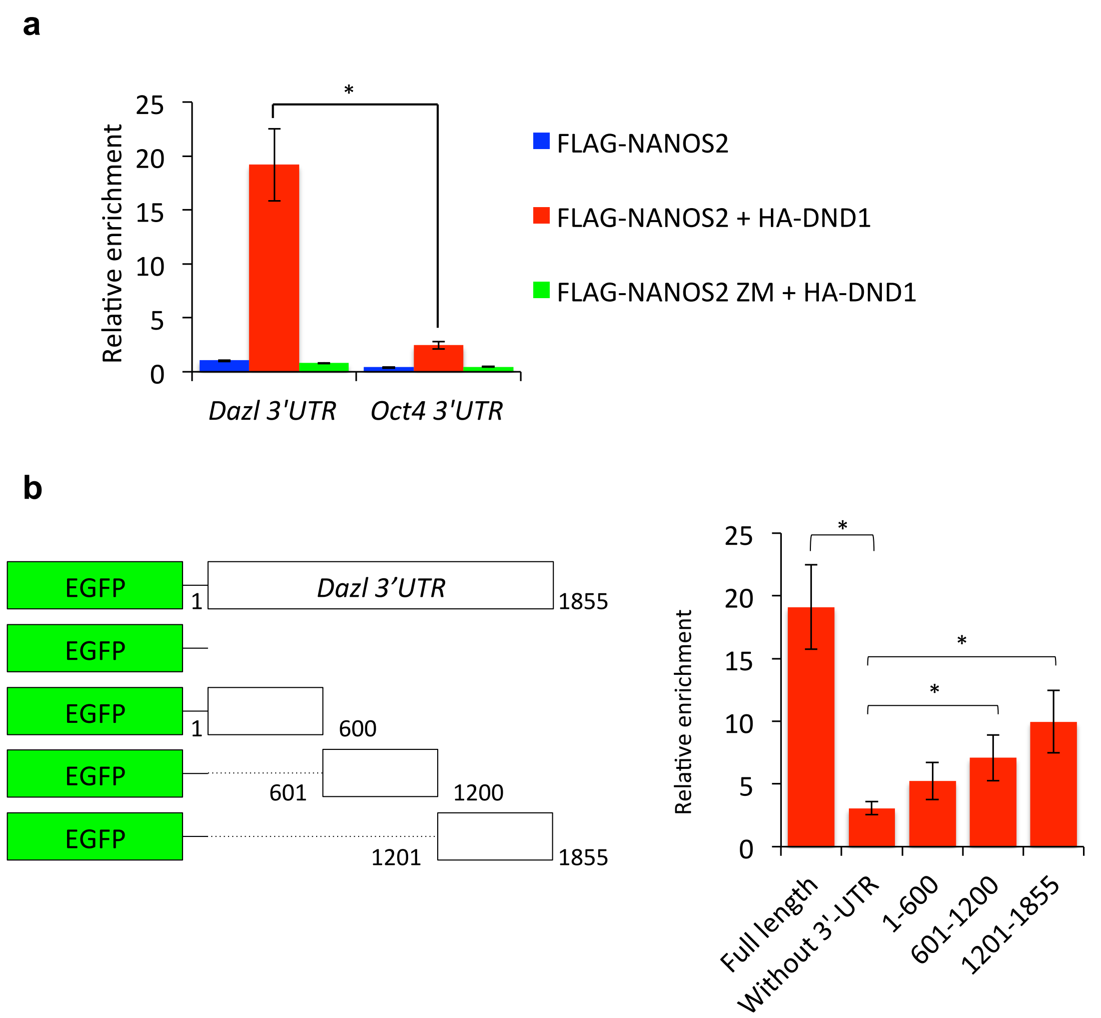

Supplementary Fig. 4. Association of NANOS2 with *Dazl* mRNA in cultured cells

(**a**) RIP followed by RT-qPCR analysis of EGFP reporter carrying *Dazl* or *Oct4* 3′ UTR

in NIH3T3 cells. Transfection of expression vectors together with EGFP reporter was done as follows; single transfection for FLAG-NANOS2 (blue), co-transfection for FLAG-NANOS2 and HA-DND1 (red), and co-transfection for FLAG-NANOS2 ZM which carries amino acid substitution in zinc finger domain of NANOS2 and HA-DND1 (green). RIP was performed by using anti-FLAG antibody and qPCR was carried out using primer set amplifying EGFP. (**b**) Mapping of NANOS2-binding region in *Dazl* 3′ UTR. The *Dazl* 3′ UTR was divided into 3 fragments (1-600, 601-1200, and 1201-1855), and co-transfected with vectors for FLAG-NANOS2 and HA-DND1. RIP and RT-qPCR were performed as in **(a)**. Data of full length *Dazl* 3′ UTR is same in **(a)**. (**a, b**) The y-axis as Fig. 1g ($n = 3$). Error bars, ±SD. Significance levels of changes are indicated (two-tailed Student's *t*-test; $^*p < 0.05$).

Supplementary Figure 5

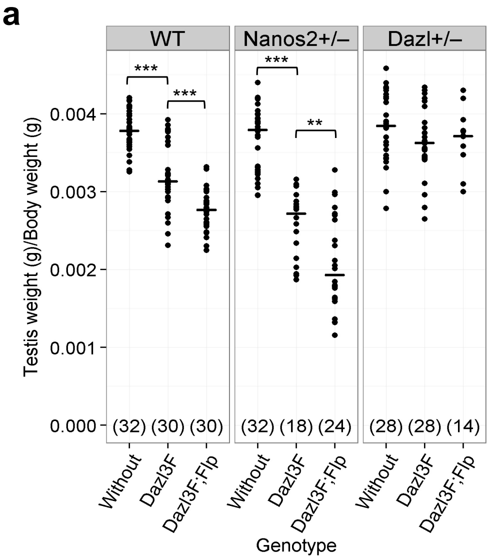

b

Tukey's HSD, P-value

	WT vs *Nanos2*$^{+/-}$	WT vs *Dazl*$^{+/-}$
Without Tg	0.4696	0.8318
Dazl3F	1.4e-05	0.0018
Dazl3F;Flp	1.1e-06	4.0e-07

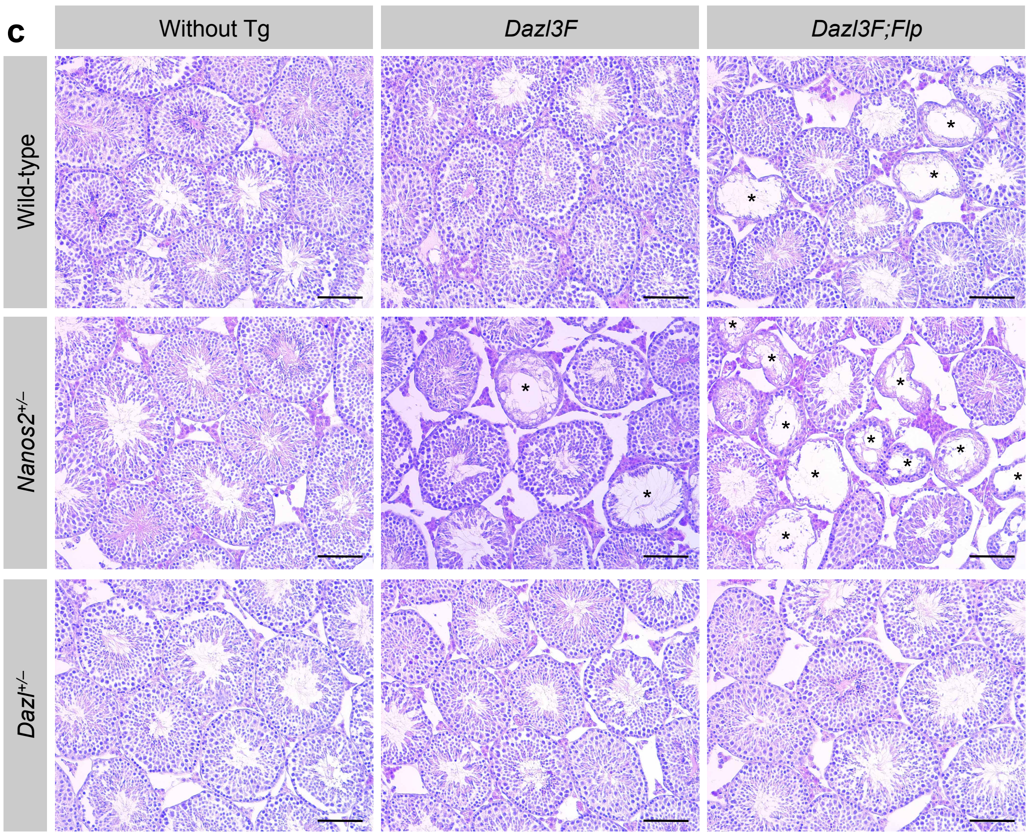

Supplementary Fig. 5. Genetic interaction between *Nanos2* and *Dazl* (a) Relative testis weights at 10 weeks after birth (10W) are shown as dot plots. Individual panels

are labelled at the top (wild-type [WT], *Nanos2*$^{+/–}$, and *Dazl*$^{+/–}$) to indicate the genetic backgrounds into which the transgenic allele (Tg) was introduced. The numbers of testes examined are indicated in parentheses at the bottom. Significance levels of changes are indicated (Tukey's multiple comparison test, ***p < 0.0005, **p < 0.005). (**b**) Statistical analysis (Tukey's honestly significant difference, HSD, test) of the relative testis weight in panel **a** between WT and *Nanos2*$^{+/–}$ or *Dazl*$^{+/–}$ mice. (**c**) Hematoxylin and eosin staining of 10W testes with different genetic backgrounds. Seminiferous tubules lacking spermatogenic cells and tubules showing defective spermatogenesis are indicated by asterisks. On the WT background, such tubules were observed in transgenic testes when the 3′ UTR was removed (upper right panel). However, such tubules were observed in transgenic testes carrying the 3′ UTR when the transgene was introduced onto the *Nanos2*$^{+/–}$ background (middle centre panel). The number of such tubules was increased when the 3′ UTR was removed (middle right panel). In contrast, no tubules showing defective spermatogenesis were observed in transgenic testes when the transgene was introduced onto a *Dazl*$^{+/–}$ background (bottom panels). Scale bars = 100 μm.

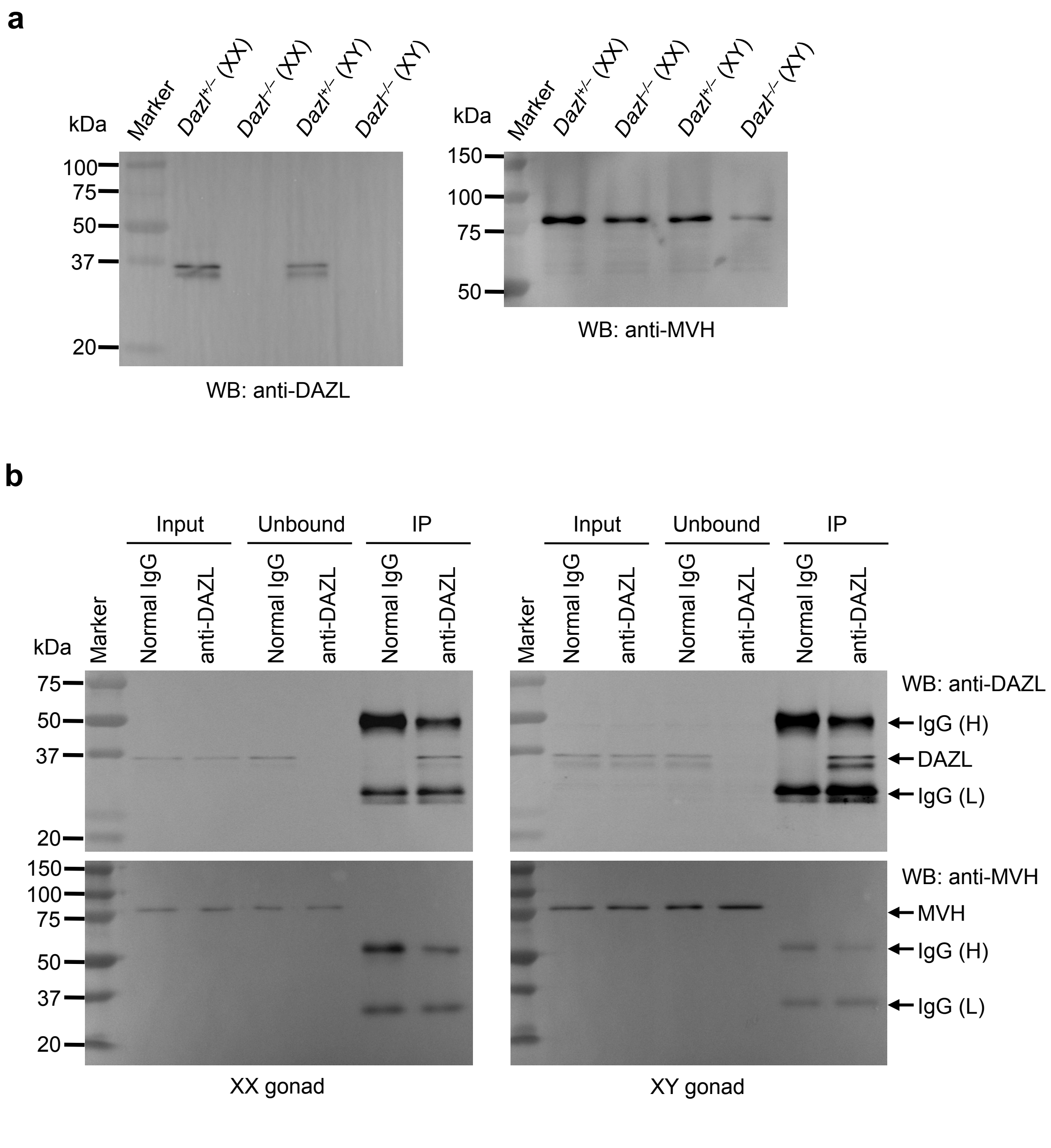

Supplementary Fig. 6. RNA-immunoprecipitation (RIP) assays of DAZL (a)

Western blot (WB) analysis showing that the anti-DAZL antibody used for the RIP

assay specifically recognizes DAZL protein. Whole cell lysates of *Dazl*$^{+/-}$ and *Dazl*$^{-/-}$ XX and XY gonads at E14.5 were reacted with anti-DAZL (left) and anti-MVH (murine *vasa* homolog; right) antibodies. (**b**) WB analysis showing efficient and specific precipitation of DAZL protein in RIP assays. Cellular extracts from XX and XY gonads at E14.5 were reacted with either normal rabbit immunoglobulin G (IgG) or anti-DAZL antibodies, which had been pre-incubated with protein G-conjugated magnetic beads. Note that DAZL signals were detected in the immunoprecipitate (IP) but not in unbound fractions, while MVH signals were detected in unbound fractions but not in the IP. H and I in parentheses indicate the heavy and light chains of IgG, respectively.

Supplementary Figure 7

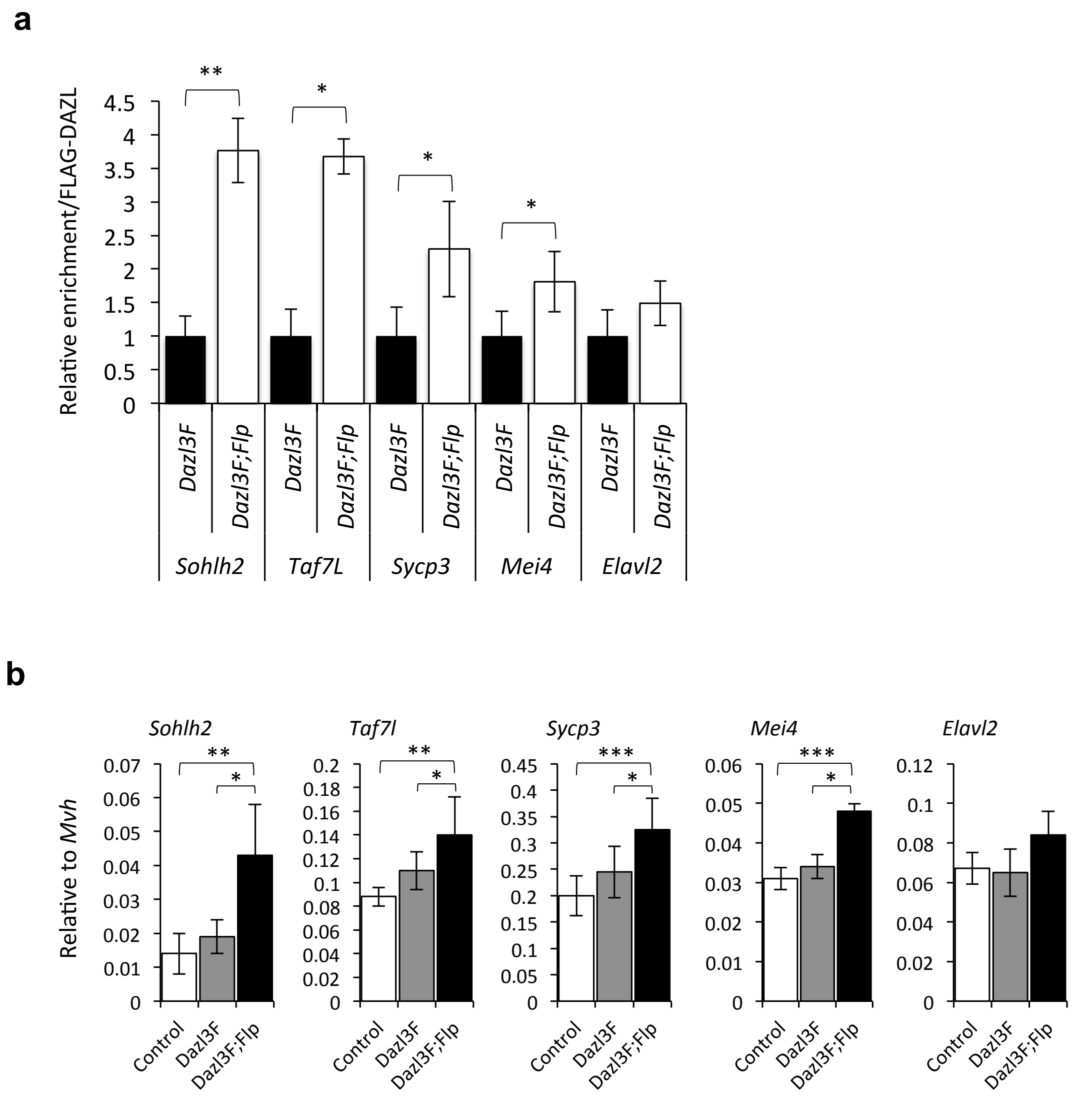

Supplementary Fig. 7. The effect of excess DAZL on target RNA binding and stability (**a**) RIP was performed with anti-FLAG antibody using *Dazl3F* and

Dazl3F;Flp XY gonads at E14.5. The relative enrichment of selected DAZL-associated mRNAs in IPs was measured by qPCR. To compare the amounts of the precipitated mRNAs in *Dazl3F* and *Dazl3F;Flp* gonads, sample volumes for cDNA synthesis were normalised based on the amounts of immunoprecipitated FLAG-DAZL protein. Error bars, ±SD ($n = 3$). Significance levels of changes are indicated (Mann-Whitney *U*-test; $**p < 0.005$, $*p < 0.05$). (**b**) Expression levels of the selected DAZL-associated mRNAs in XY gonads at E15.5 by RT-qPCR. The data of *Scyp3* was same as in figure 2a. The y-axis shows the expression level relative to *Mvh*. Error bars, ±SD ($n = 3–8$). Significance levels of changes are indicated (two-tailed Student's *t*-test; $***p < 0.0005$, $**p < 0.005$, $*p < 0.05$).

Supplementary Figure 8

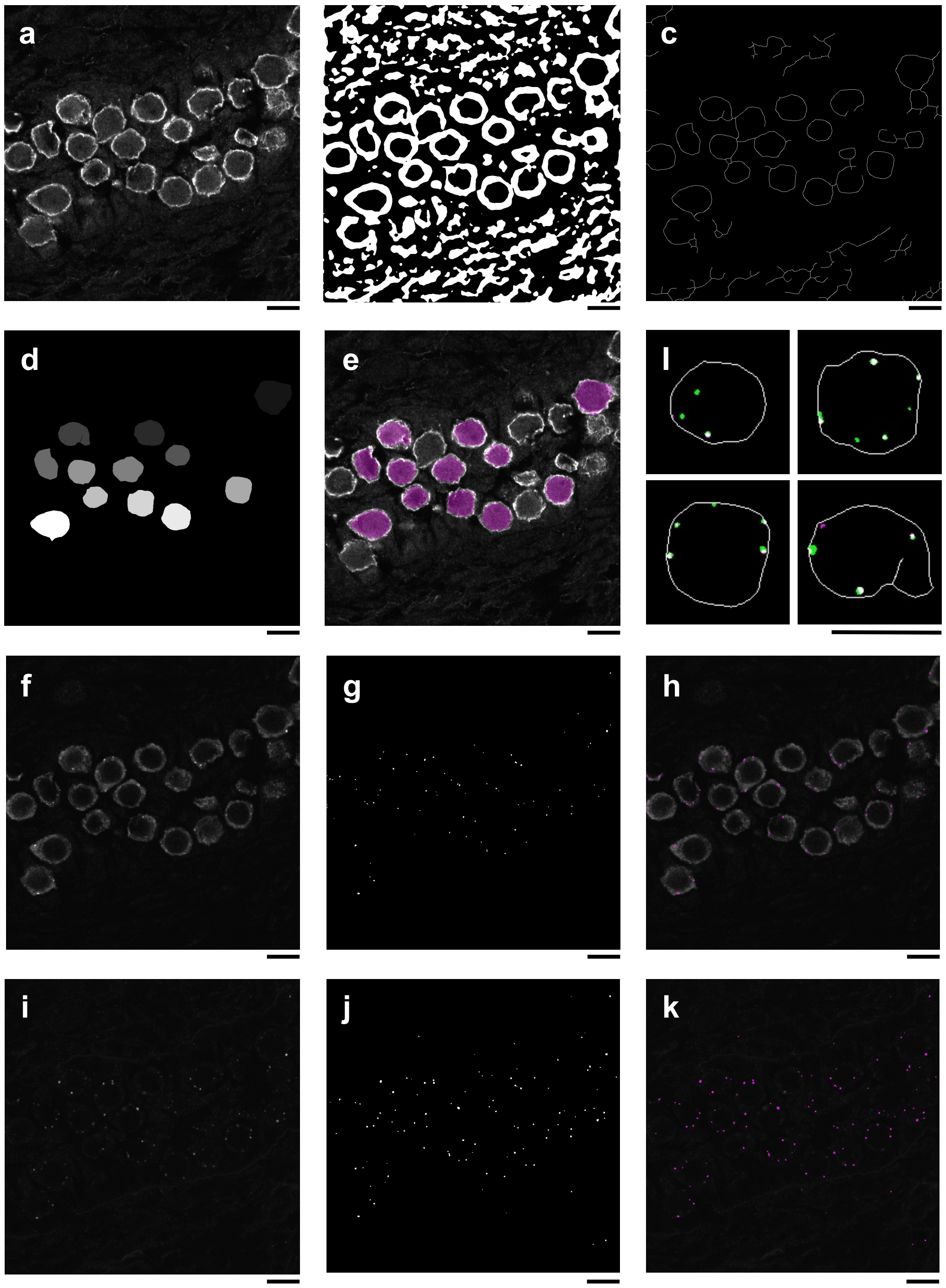

Supplementary Fig. 8. Image analysis of granular structures in XY germ cells (a-e)

Segmentation of germ cells. (**a**) CDH1/E-cadherin staining. (**b**) Binary image of **a** after

applying a Gaussian blur filter and local thresholding. (**c**) Skeleton image of **b** after size filtering. (**d**) Segmented germ cells after filling holes and subtracting the skeleton in **c**. Each grey-scale image outline indicates a different object. (**e**) Germ cell masks (magenta) overlaid on the original CDH1 image. Note that cells with discontinuous edges were not recognised. (**f-k**) Recognition of granular objects using immunostaining for NANOS2 (**f**-**h**) or DCP1a (**i**-**k**). (**g** and **j**) Granules detected by applying the differences in Gaussian blur filter images followed by local thresholding and size selection. (**h** and **k**) Detected granules were overlaid on the original images. (**l**) Examples of individual germ cells with granular structures positive for NANOS2 (magenta) and DCP1a (green). (**a-l**) Scale bars = 10 μm.

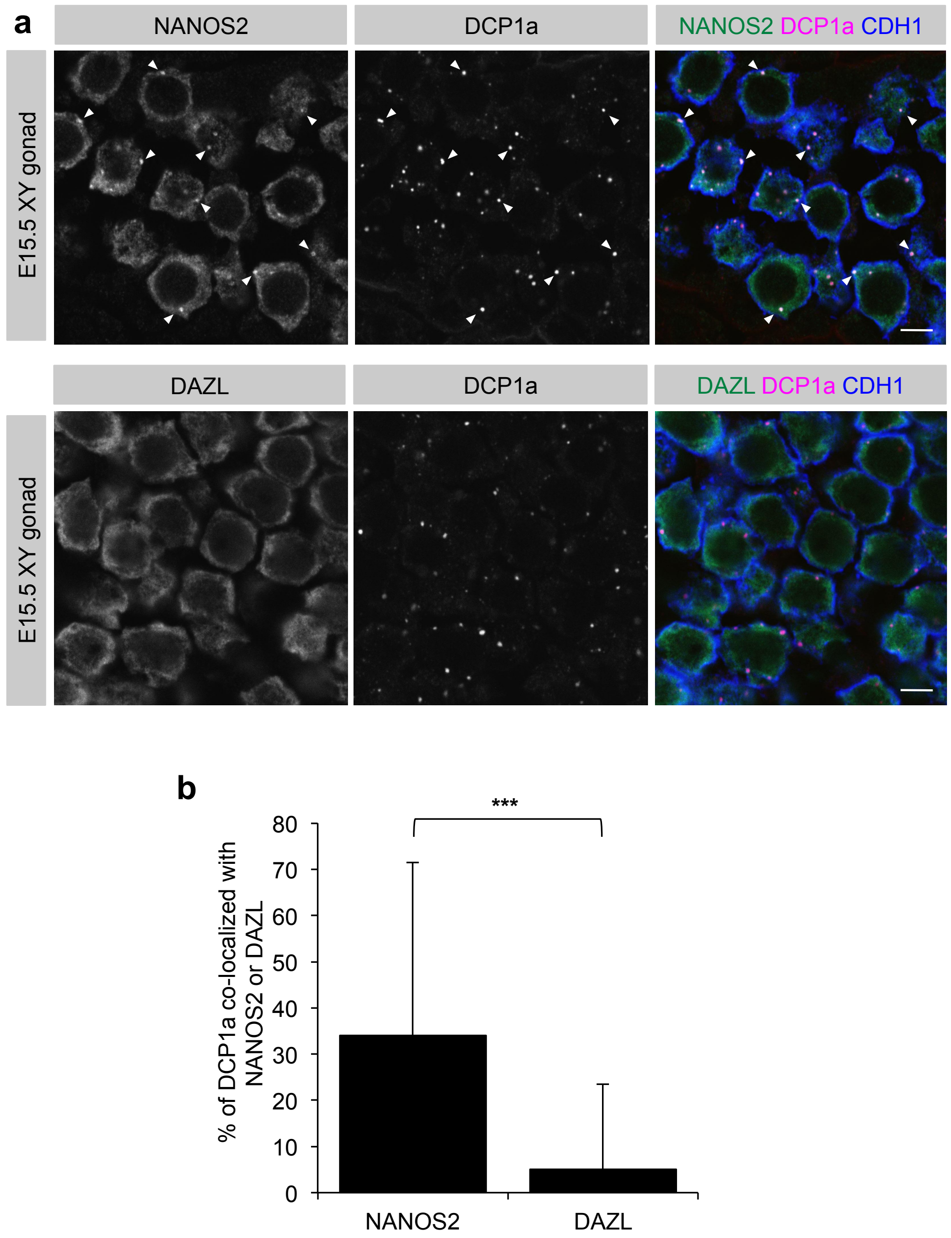

Supplementary Fig. 9. Co-localisation analysis of NANOS2 and DAZL with P-body in XY germ cells Co-localisation of NANOS2 and DAZL with DCP1a was analysed by

immunofluorescence staining and image analysis. (**a**) XY gonads at E15.5 were stained by either anti-NANOS2 or anti-DAZL antibodies, together with anti-DCP1a and anti-CDH1 antibodies. Arrowheads indicate granules merged with DCP1a staining. Scale bars = 5 µm. (**b**) Proportion of DCP1a granules co-localised with NANOS2 or DAZL granules in wild-type XY germ cells at E15.5. n = 1228 (NANOS2) or 1285 (DAZL). Significance of changes is indicated (Mann-Whitney U-test, $***p < 0.0005$).

Supplementary Figure 10

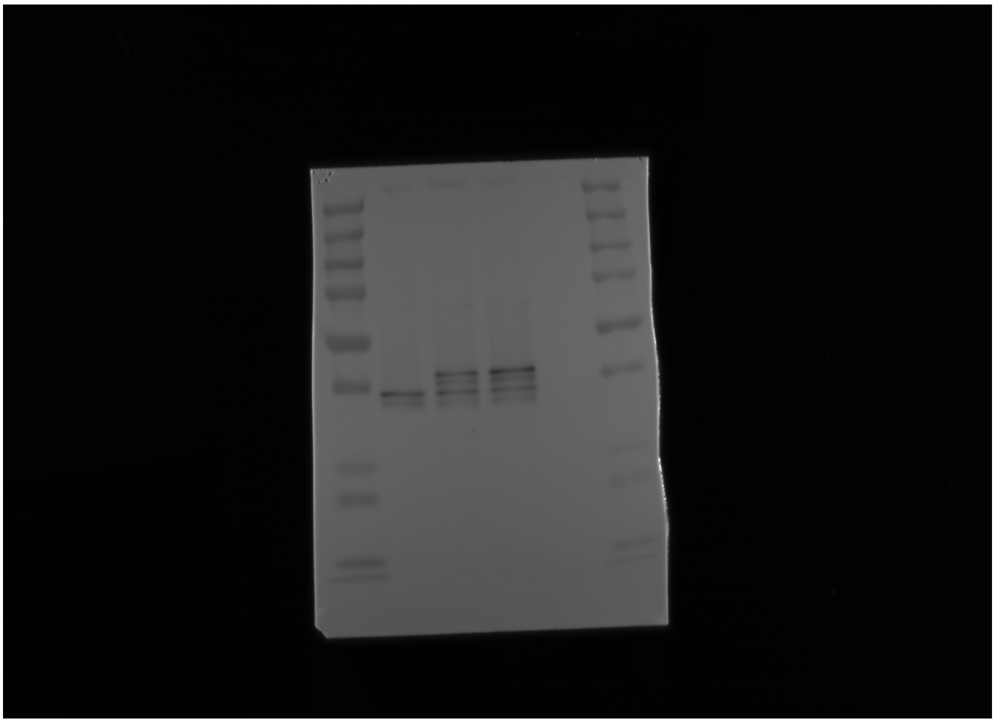

Supplementary Fig. 10. A representative image of uncropped western blot scan A western blot image of E15.5 XY gonad extract reacted with anti-DAZL antibody in Fig. 1e.

Supplementary Table 1. Selected candidates of NANOS2 target mRNAs

Probe ID	GeneSymbol	EntrezGeneID	Chromosome	Fold decrease in Nanos2-expressing XX gonads (log2)	Fold increase in Nanos2-null XY gonads at E14.5 (log2)	Fold increase in Nanos2-null XY gonads at E15.5 (log2)	Fold enrichment (log2)
A_52_P462296	*Cpeb1*	12877	7	-0.7011	0.9851	1.5189	3.4619
A_51_P512293	*Gm7789*	665798	7	-0.9878	1.826	1.2618	2.0471
A_51_P248687	*Hormad2*	75828	11	-0.7429	1.1576	2.3353	2.8941
A_51_P353776	*Gjb3*	14620	4	-0.6676	1.6673	0.8297	2.3932
A_51_P246060	*Dazl*	13164	17	-0.6161	0.9967	1.4923	2.262
A_52_P537571	*Trap1a*	22037	X	-1.0249	1.0564	1.4652	2.695
A_51_P143712	*Tdh*	58865	14	-0.9802	3.0258	2.9001	2.3914
A_51_P219849	*Alg13*	67574	X	-0.727	0.8669	1.4015	2.8173
A_51_P294233	*Nanog*	71950	6	-0.7422	1.5028	1.7023	2.5945
A_52_P664899	*Elavl2*	15569	4	-0.6527	1.0132	0.9856	3.1132
A_51_P134835	*Elavl2*	15569	4	-0.8538	0.6511	1.0101	2.0163
A_51_P333594	unknown			-1.0606	1.7361	1.5689	2.2497
A_51_P404992	*Efhc2*	74405	X	-0.7631	0.8733	1.6766	3.4421
A_52_P127184	*Efhc2*	74405	X	-0.703	0.9949	1.4661	3.4213
A_51_P132483	*1700013H16Rik*	75514	X	-1.4184	1.6094	4.1517	3.2829
A_51_P486681	*Ap3b2*	11775	7	-0.5976	1.7133	1.8511	2.3761
A_51_P378699	*Sohlh2*	74434	3	-0.9815	2.1789	3.0026	2.0199
A_51_P418737	*Rbm33*	381626	5	-0.647	1.2261	1.093	3.5694
A_51_P214747	*Parp12*	243771	6	-0.6077	0.9219	1.1952	2.3916

Supplementary Table 2. Primers used for RT-qPCR

Gene symbol	Sense (5' to 3')	Antisense (5' to 3')
Arrdc4	CACTGAGCGTCAAGATCGAGAG	CTTTCCACTAGCCAAGTACGTC
Coq10b	TATCCAAGAACTTGTACCTTGG	CTTCATCGAAGAACAGAGTAGC
Dazl (Flag)	TGGACCGAAGCATACAGACAGTGGT	CACCGTCATGGTCTTTGTAGTC
Dazl (unspliced)	TGGACCGAAGCATACAGACAGTGGT	GTTTAGAAAATATGTACACTATAGG
Dazl (spliced)	TGGACCGAAGCATACAGACAGTGGT	TGATCAGATTTAAGCACTGCCCGAC
Dmc1	CCCTCTGTGTGACAGCTCAAC	GGTCAGCAATGTCCCGAAG
Dnmt3L	GCTATGCGGGTGTGGAGCAAC	TCACCAGGAGGTCAACTTTCG
Elavl2	GATGCAAACTTATACGTCAGCG	AAACCCTACACCCCTTGATATG
Gapdh	ACCACAGTCCATGCCATCAC	TCCACCACCCTGTTGCTGTA
Hormad2	CTACTGAGATAGCTCATCAGGG	GACTGTCACTGGTTCGCTGACC
Lhx8	ATGGCTTATTCTGCCTACGTAC	ATGACTTATTGGCAGCTGGGTC
Mei4	AGGCTCCCTGGAACCAGGTATG	CACCACCTTGATTTCCAACTGG
Miwi2	CCCTGGAAAGCCGCTGGCAC	GGTCTCTTGAGGACCGCCCCA
Mvh (unspliced)	GTTGAAGTATCTGGACATGATGCAC	CTGTCATTCAAGGATCCTCAGC
Mvh (spliced)	GTTGAAGTATCTGGACATGATGCAC	CGAGTTGGTGCTACAATAATACACTC
Nanos2	CCAGCTGAAGACGCCTGAAG	AGAGACTGCTGACTGCTGTTG
Sohlh2	CTTTGGAGGGAGCAGTGAGAG	GTGCAGTGGGTGGCAAATAAG
Stra8	CCTAAGGAAGGCAGTTTACTCCCAGTC	GCAGGTTGAAGGATGCTTTGAGC
Sycp1	ATGAGCGGGAAGAAACCAGA	GGCATTCTCAGCTTGCACAC
Sycp3	AGATCTGTCTGGTTCAGAAGAAG	CAGCATATTCTGTACTTCACC
Taf7l	CACTCCAGGAATCTCACAGATG	CACATCATTACTGTTGCTGCCAG
Tdrd9	TACGTGCTAGACCACTACACC	AACGCTCCTTACTGATCCACC
Trim6	TCGAGCACCGGATCTGAGGAAG	AGCCGTCTGTGGATTCAGTGTC
Tcfap2c	CCACGCGGAAGAGTATGTTGTT	TATGTTCGGCTCCAAGACCTGG
GFP	GCAAGCTGACCCTGAAGTTC	GGCTGAAGCACTGCACGCCG
